# Dental plaque biofilm transforms host-derived β_2_-microglobulin into polymorphic fibrils for integration into the biofilm matrix

**DOI:** 10.1016/j.bioflm.2025.100331

**Published:** 2025-11-16

**Authors:** Taiki Mori, Eisuke Domae, Mariko Hanaoka, Takeshi Into

**Affiliations:** Department of Oral Microbiology, Division of Oral Infection Health Sciences, Asahi University School of Dentistry, Gifu, Japan

**Keywords:** Dental plaque, Biofilm, Biofilm matrix, β_2_-microglobulin, Amyloid fibril, *Streptococcus mutans*, Host-derived proteins

## Abstract

Dental plaque is a polymicrobial biofilm formed on tooth surfaces despite continuous exposure to variable host-derived antimicrobial factors. To date, the underlying mechanisms remain nebulous. This study aimed to determine whether dental plaque biofilms affect the major salivary antimicrobial protein β_2_-microglobulin (B2M). Immunostaining of human dental plaque specimens with an anti-B2M antibody revealed that B2M exists as elongated fibers, punctate structures, and amorphous aggregates. Fractionation of dental plaque suspensions revealed that B2M was present in both the soluble and insoluble fractions. B2M, which forms insoluble fibrils associated with dialysis-related amyloidosis, exhibited comparable fibril-forming properties in dental plaque. Immunostaining with a developed anti-B2M amyloid fibril antibody showed that fibrillar B2Ms (fB2Ms) were distributed throughout the dental plaque specimens. *In vitro* experiments using purified B2M demonstrated that environmental factors characteristic of dental plaque, specifically phosphate ions, bacterial short-chain fatty acids (acetic, butyric, lactic, and propionic acids), and divalent calcium and magnesium ions, significantly promoted fB2M formation. In a *Streptococcus mutans* biofilm model, native B2M transformed into fibrils only in the presence of these environmental factors, resulting in the loss of its antimicrobial activity and its incorporation into the biofilm matrix. The preformed fB2Ms increased *S. mutans* biofilm growth, decreased biofilm adhesion, and transformed the biofilm matrix architecture from a membranous to a reticulated organization, potentially facilitating biofilm dissemination. Dental plaque biofilms employed a specialized “molecular hijacking” strategy to counteract host defense mechanisms and ensure persistence through fibrillation. Our findings provide novel insights into biofilm pathogenesis, host-microbe interactions, and potential plaque control approaches.

## 1 Introduction

Dental plaque, a specialized polymicrobial biofilm that forms on tooth surfaces [1,2], exhibits remarkable microbial diversity and harbors hundreds of bacterial species, ranging from facultative anaerobes such as *Streptococcus* species to strict aerobes and obligate anaerobes [3]. The extracellular matrix of dental plaque biofilms is primarily composed of polysaccharides, particularly glucans and fructans, synthesized from dietary sucrose, and other macromolecular components, including proteins, nucleic acids, and lipids [4]. Within the dental plaque biofilms, bacteria produce short-chain fatty acids (SCFAs) by metabolizing dietary or salivary carbohydrates [[5], [6], [7]]. This creates and maintains an acidic microenvironment, which significantly influences the biofilm ecology and pathogenic potential, such as dental caries and periodontitis. Dental plaque formation also occurs in the presence of saliva, which provides abundant calcium and phosphate ions (Ca^2+^ and PO_4_^3−^) that are essential for tooth remineralization [4,8] and become integral constituents of the biofilm. Additionally, the plaque matrix contains various ionic species, including chloride and bicarbonate anions, as well as sodium and magnesium cations derived from salivary and gingival crevicular fluid sources [8].

Dental plaque biofilms are continuously exposed to components in saliva and gingival crevicular fluid, both of which contain varied antimicrobial factors, such as secretory IgA, lysozyme, lactoferrin, and LL-37, which typically hinder biofilm growth [4,9,10]. Despite the presence of these host defense factors, dental plaque biofilms develop and persist in the oral cavity, suggesting the existence of specialized strategies to resist or neutralize these factors. We have previously reported that the antimicrobial peptide LL-37 binds to genomic DNA released from plaque bacteria, forming insoluble complexes that diminish the antimicrobial activity of LL-37 upon incorporation into the plaque matrix as structural components [11]. This mechanism may demonstrate how plaque biofilms protect themselves from host defenses while also repurposing these factors as beneficial building blocks. However, the mechanisms underlying this neutralization and incorporation strategy remain largely unexplored, representing a critical gap in understanding host-biofilm interactions and biofilm pathogenesis.

β_2_-Microglobulin (B2M), a low-molecular-weight protein (11.8 kDa) with 99 amino acids, is ubiquitously produced in all nucleated cells, except for erythrocytes [12]. Its highly stable structure, characterized by seven small β-strands forming a barrel-like configuration, supports its role as a critical component of MHC class I molecules, stabilizing antigen-binding ability and aiding endogenous antigen presentation on cell surfaces [13,14]. When dissociated from MHC class I molecules, B2M is released into extracellular fluids, including blood, at a concentration range of 1–3 μg/mL in healthy individuals [15]. Free B2M exhibits antimicrobial properties through two concentration-dependent mechanisms: at lower concentrations, it exerts a bacteriostatic effect by indirectly damaging the cell membranes of pathogens, as reported in *Listeria monocytogenes* and *Escherichia coli*, thereby dissipating the membrane potential [16]. In contrast, at higher concentrations under mildly acidic conditions, B2M exerts a bactericidal effect against *Pseudomonas aeruginosa* via direct binding to bacterial surfaces [17]. These findings suggest that B2M is a multifunctional protein with roles beyond antigen presentation and is a key player in the host's innate immune system against infections.

In the oral cavity, B2M is a major salivary protein, with an average concentration of approximately 2.1 μg/mL. It is present in gingival crevicular fluid, ensuring its widespread distribution throughout the oral environment [18,19]. This ubiquitous presence suggests that B2M plays a significant role in shaping oral bacterial communities and dental plaque biofilm formation. Pioneering reports in the 1970s and the 1980s demonstrated its potential functions in oral microbiology and direct binding to the surfaces of various oral streptococci, including *Streptococcus mutans* [20,21]. Furthermore, B2M was found to induce the aggregation of *S. mutans* in the presence of Ca^2+^ [22], indicating its role as a salivary agglutinin that promotes bacterial clustering. Despite these findings, the precise role of B2M in oral health and disease remains poorly understood.

Previous research primarily focused on the effects of purified B2M on cultured oral streptococci, leaving its *in vivo* role and impact on dental plaque biofilms unclear. To address this gap, we investigated the role of B2M in dental plaques using human specimens. Unexpectedly, our immunochemical analyses revealed that B2M exists within dental plaque as microscopic-scale structures. Further investigation demonstrated that these structures represented polymorphic fibrils. This study provides the first comprehensive evidence of the dynamic interplay between dental plaque biofilms and host-derived B2M in the oral cavity. Our findings highlight how host defense factors can be co-opted and functionally altered by dental plaque biofilms, offering novel insights into oral microbial pathogenesis.

## 2 Results

### 2.1 Dental plaque biofilms contain B2M as insoluble structures

**Fig. 1:**
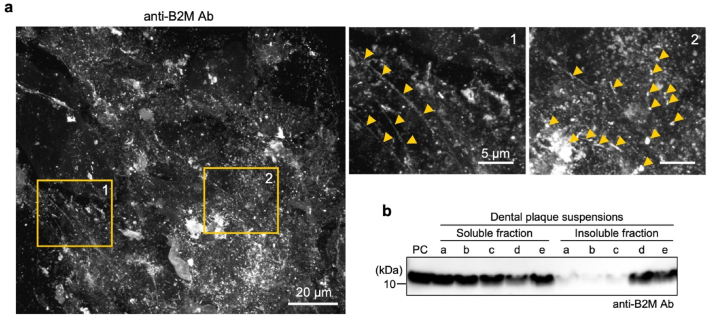
β_2_-Microglobulin (B2M) exists as insoluble components exhibiting diverse morphologies in dental plaque. **(a)** Confocal microscopy images of dental plaque specimens stained using an anti-B2M antibody (Ab). Left image shows an overview (Scale bar: 20 μm). The areas outlined in orange (1 and 2) in the left panel are magnified and displayed in the middle image (1), highlighting structures with long fibrillar morphologies (orange arrowheads). The right image (2) highlights structures with punctate or short fibrillar forms of various sizes (orange arrowheads). Scale bars: 5 μm. **(b)** Western blot analysis of B2M in the soluble and insoluble fractions of five dental plaque suspensions (a, b, c, d, and e) using an anti-B2M Ab. PC, B2M positive control (15 ng). (For interpretation of the references to colour in this figure legend, the reader is referred to the Web version of this article.)

We hypothesized that dental plaque biofilm formation proceeds by inactivating the antimicrobial proteins present in the saliva or gingival crevicular fluid, while simultaneously incorporating them into the biofilm. We focused particularly on B2M. First, to investigate the presence of B2M in dental plaque biofilms, five human dental plaque specimens were immunostained with a commercially available anti-B2M antibody (Ab) and observed via confocal microscopy. Intriguingly, B2M was observed as microscopic-scale structures with several morphologies such as elongated fibrils, punctate aggregates, and amorphous deposits (Fig. 1a). These structures were detected in two specimens and were not uniformly present in the dental plaque, but appeared locally abundant.

In the soluble fraction of the specimens, immunoblot analysis revealed that B2M was abundantly present. In contrast, in the insoluble fraction, the presence of B2M showed individual variability, being detected in two individuals but absent in the others (Fig. 1b). These findings indicate that B2M is incorporated into dental plaque biofilms as insoluble structures that are possibly transformed by an individual-specific plaque microenvironment.

### 2.2 B2M undergoes polymorphic fibril formation in dental plaque biofilms

**Fig. 2:**
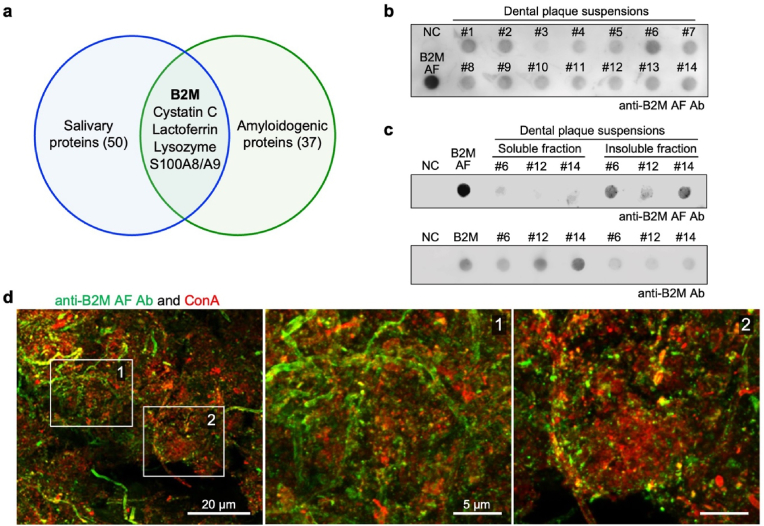
β_2_-microglobulin (B2M) exists as fibrils exhibiting diverse morphologies in dental plaque. (**a)** Venn diagram showing the overlap between 50 human salivary proteins (Table S1) and 37 amyloidogenic proteins (Table S2); the intersection of these sets includes B2M, cystatin C, lactoferrin, lysozymes, and S100A8/A9, all of which function as antimicrobial factors. **(b)** Dot blot analysis of fibrillar B2Ms (fB2Ms) in dental plaque specimen suspensions acquired from 14 subjects (Table S3) using an anti-B2M AF Ab. NC, negative control (PBS); B2M AF, fibrillized B2M positive control (20 ng). **(c)** Dot blot analysis of fB2M (upper) and B2M (lower) in the soluble and insoluble fractions of three dental plaque suspensions (#6, #12, and #14) using an anti-B2M AF Ab or anti-B2M Ab. NC, negative control (PBS); B2M AF, fibrillized B2M positive control (20 ng); B2M, B2M positive control (10 ng). **(d)** Confocal microscopy analysis of dental plaque specimens immunofluorescently stained with an anti-B2M AF Ab (green) and Concanavalin A (ConA; red, indicating extracellular glycoconjugates). Scale bar: 20 μm. The areas outlined in white (1 and 2) in the left image are magnified and displayed in the middle (1) and right (2) images. Scale bars: 5 μm. (For interpretation of the references to colour in this figure legend, the reader is referred to the Web version of this article.)

B2M, one of the major salivary proteins (Table S1), is also known as an amyloidogenic protein (Table S2) [10,23]. As shown in Fig. 2a, B2M shares these properties with five other proteins, all of which possess antimicrobial properties [9]. In humans, amyloidogenic proteins, including B2M, are intrinsically prone to misfolding under certain conditions, ultimately aggregating into amyloid fibrils (AFs)—the insoluble polymeric assemblies characterized by cross-β-sheet quaternary structures [24]. Such transformation is implicated in diverse proteinopathies, in which tissue-deposited AFs disrupt cellular homeostasis and promote disease progression [23]. Meanwhile, microbial communities also produce AFs and utilize them as structural components of biofilms [25]. Furthermore, microbes can generate non-amyloid fibrils, including those with cross-α structures [26,27], which may also influence biofilm formation. Hence, dental plaque biofilms might provide a favorable environment for the production of fibrillar proteins. Accordingly, we investigated whether the insoluble B2M aggregates observed in human dental plaque specimens represent changes consistent with AF formation.

To specifically detect fibrillar B2M (fB2M) in dental plaque biofilms, we developed an anti-B2M AF polyclonal Ab by immunizing mice with B2M AF that was artificially generated using purified B2M according to an established method for B2M amyloidogenesis [28]. This Ab demonstrated high specificity for fB2Ms (Fig. S1) and reacted with 10 out of the 14 tested dental plaque specimens in dot blot analyses (Fig. 2b). The anti-B2M AF Ab exhibited reactivity toward the insoluble fractions from dental plaque specimens, even in those with low insoluble B2M levels, while showing minimal cross-reactivity with the soluble fractions (Fig. 2c, upper panel). Conversely, a conventional anti-B2M Ab primarily reacted with the soluble fractions (Fig. 2c, lower panel). These samples provided sufficient quantities for experimentation and were subsequently assessed to determine whether fractionation into soluble and insoluble fractions would yield detection intensities comparable to those observed in the dot blot analyses.

Furthermore, immunostaining using the anti-B2M AF Ab detected diverse structural morphologies, including elongated fibrils, fragmented fibrils, punctate aggregates, and amorphous deposits (Fig. 2d). Partial co-localization was also observed between signals for the anti-B2M AF Ab and the conventional anti-B2M Ab (Fig. S3), suggesting that the detection of these structures was not attributable to non-specific reactions.

To further demonstrate the presence of fibrillar structures within dental plaque, a commercially available anti-AF Ab raised against human islet amyloid polypeptide (IAPP)-derived Afs was utilized, as proteins with distinct fibrillar morphology have not been previously demonstrated in dental plaques. The dental plaque specimens showed positive reactivity with the anti-IAPP AF Ab in dot blotting (Fig. S2a), and this Ab reacted solely with insoluble fractions (Fig. S2b). Immunostaining of the specimens with the anti-IAPP AF Ab similarly detected locally abundant fibrillar structures (Fig. S2c).

These findings collectively demonstrate that B2M undergoes fibrillation, forming insoluble, structurally polymorphic fibrils associated with dental plaque biofilms.

### 2.3 Identification of the dental plaque biofilm-specific drivers of B2M fibrillation

**Fig. 3:**
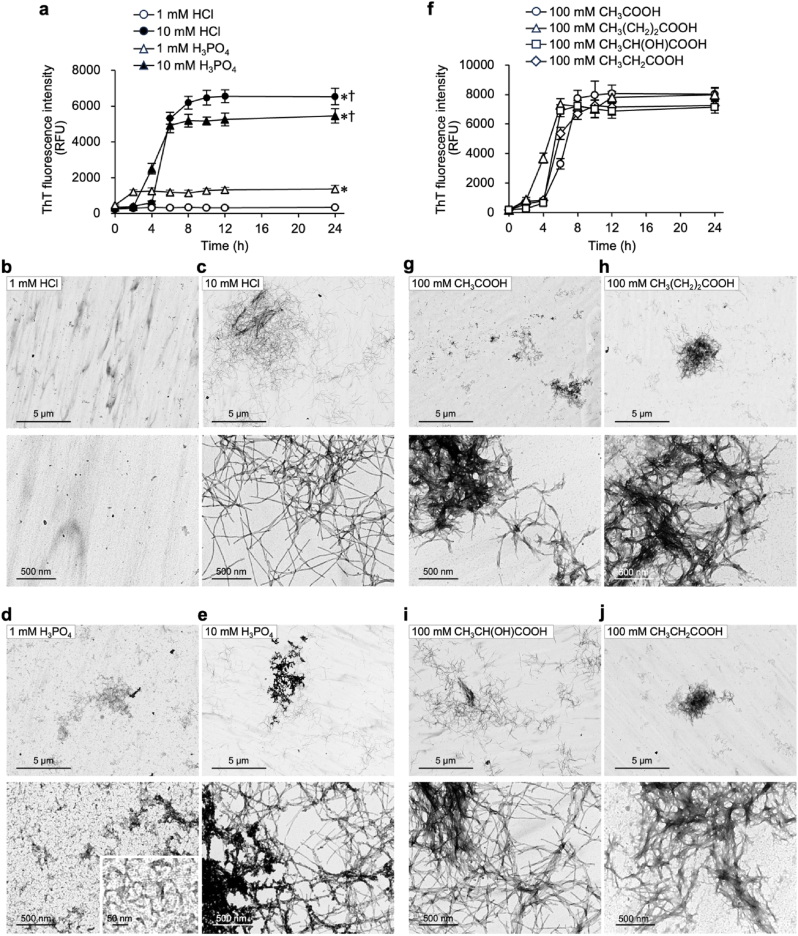
β_2_-microglobulin (B2M) forms diverse fibrils in the presence of H_3_PO_4_ and short-chain fatty acids (SCFAs). (a) *In vitro* fibrillar B2M (fB2M) formation under acidic conditions (induced by HCl or H_3_PO_4_). B2M (500 μg/mL) was incubated at 37 °C for 0–24 h in the presence of 150 mM NaCl, either with 1 mM HCl (open circle), 10 mM HCl (closed circle), 1 mM H_3_PO_4_ (open triangle), or 10 mM H_3_PO_4_ (closed triangle). ThT fluorescence intensity (relative fluorescence units; RFU) reflecting fibril formation is presented as the mean ± SD (n ≥ 3). ∗*P* < 0.01 compared to 1 mM HCl at 24 h; ^†^*P* < 0.01 compared to 1 mM H_3_PO_4_ at 24 h. **(b**–**e)** Transmission electron microscopy (TEM) images of B2M treated with 1 mM HCl (b), 10 mM HCl (c), 1 mM H_3_PO_4_ (d), or 10 mM H_3_PO_4_ (e) in the presence of 150 mM NaCl. Images were acquired at magnifications of × 2000 (upper) and × 15,000 (lower). Scale bars: 5 μm and 500 nm, respectively. The inset in the image (d) was acquired at a magnification of × 60,000 (scale bar: 50 nm). **(f)***In vitro* fB2M formation under acidic conditions induced by bacterial SCFAs. B2M (500 μg/mL) was incubated at 37 °C for 0–24 h either with 100 mM of CH_3_COOH (open circle), CH_3_(CH_2_)_2_COOH (open triangle), CH_3_CH(OH)COOH (open square), or CH_3_CH_2_COOH (open diamond), in the presence of 150 mM NaCl. ThT fluorescence intensity (RFU) reflecting fibril formation is expressed as the mean ± SD (n ≥ 3). **(g**–**j)** TEM images of B2M treated with 100 mM of each CH_3_COOH (g), CH_3_(CH_2_)_2_COOH (h), CH_3_CH(OH)COOH (i), or CH_3_CH_2_COOH (j) in the presence of 150 mM NaCl. Images were acquired at magnifications of × 2000 (upper) and × 15,000 (lower). Scale bars: 5 μm and 500 nm, respectively.

AF formation typically follows a biphasic process: 1) In the nucleation phase, soluble monomers overcome kinetic barriers to form metastable oligomeric nuclei. 2) In the elongation phase, nuclei undergo template-directed monomer recruitment, enabling exponential fibril growth [29]. For B2M amyloidogenesis, nucleation requires specific physicochemical cues—acidic pH, anion type and anion concentration—coupled with mechanical agitation [30]. In the subsequent acid-dependent B2M AF formation process, anion concentration is more important than pH [30]. This is exemplified by *in vitro* amyloid fibrillation protocols where native B2M forms AFs following hydrochloric acid (HCl) exposure with ionic strength modulators (50–600 mM NaCl) during shaking or ultrasonication [28,29,31]. Consistent with these established protocols, we observed concentration-dependent outcomes: 1 mM HCl failed to induce Thioflavin T (ThT) fluorescence, a hallmark of β-sheet-rich assemblies, whereas 10 mM triggered progressive ThT fluorescence intensification between 4 and 6 h, plateauing at 8 h (Fig. 3a). Correlating with the ThT profile, transmission electron microscopy (TEM) showed that 1 mM HCl produced no obvious structures of B2M, while 10 mM generated homogeneous, typical elongated fibrils (Fig. 3b and c).

Using this experimental system, we investigated whether environmental factors in human dental plaque biofilms contribute to the formation of fB2M. To this end, we assessed the amyloidogenic potential of the dental plaque-associated acids. A slight increase in ThT fluorescence was induced by 1 mM phosphoric acid (H_3_PO_4_), whereas 10 mM H_3_PO_4_ triggered a significant rise in fluorescence between 4 and 6 h (Fig. 3a). TEM also revealed distinct morphological differences: 1 mM H_3_PO_4_ produced tiny worm-shaped fibrils (Fig. 3d) whereas 10 mM generated elongated fibrils along with clumpy aggregates (Fig. 3e).

We evaluated SCFAs produced by bacterial metabolism: acetic acid (CH_3_COOH), butyric acid (CH_3_(CH_2_)_2_COOH), lactic acid (CH_3_CH(OH)COOH), and propionic acid (CH_3_CH_2_COOH). At 100 mM concentrations, all SCFAs induced a marked increase in ThT fluorescence, rising significantly between 4 and 6 h and plateauing by 8 h (Fig. 3f). CH_3_COOH, CH_3_(CH_2_)_2_COOH, and CH_3_CH_2_COOH promoted heterogeneous, thick atypical fibrils that aggregated into clusters (Fig. 3g, h, j); CH_3_CH(OH)COOH formed elongated, thin typical fibrils structurally similar to those in 10 mM H_3_PO_4_ but without the clumpy aggregates observed in the latter (Fig. 3i). These findings demonstrate that anion type and concentration, rather than pH alone, drive the formation of diverse fB2M morphologies. In dental plaque biofilms, such anions include PO_4_^3−^ and bacterial SCFA anions.

The formation of B2M AFs is modulated by cation species under neutral conditions [32,33]. We examined the effects of divalent cations—Ca^2+^ and Mg^2+^—abundant in dental plaque biofilms [34]. Under H_3_PO_4_-mediated acidic conditions, 150 mM CaCl_2_ or MgCl_2_ displayed remarkably strong effects on ThT fluorescence, with magnitudes comparable to those observed with 300 mM NaCl (Fig. S4a). TEM revealed that 150 mM CaCl_2_/MgCl_2_ induced fibril aggregates organized into mesh-like structures, compared to the less organized fibrils with 150 mM NaCl (Fig. S4b–e). In contrast, under CH_3_COOH-mediated acidic conditions, 150 mM NaCl produced the highest ThT fluorescence, while 300 mM NaCl, 150 mM CaCl_2_, and 150 mM MgCl_2_ yielded marginal increases (Fig. S4f). TEM showed aggregated fibrils across all conditions (Fig. S4g–j). Notably, structures formed with 150 mM CaCl_2_, 150 mM MgCl_2_ or 300 mM NaCl were insufficiently detected by ThT fluorescence (Fig. S4f), possibly due to their insufficient ThT-binding affinity despite visible fibril aggregation. These results demonstrate that the cation type critically regulates fB2M formation and morphology in dental plaque biofilms, particularly with divalent cations (Ca^2+^/Mg^2+^) favoring fibril formation in PO_4_^3−^-rich environments.

### 2.4 S. mutans biofilm converts B2M into fibrils and incorporates them as biofilm components

**Fig. 4:**
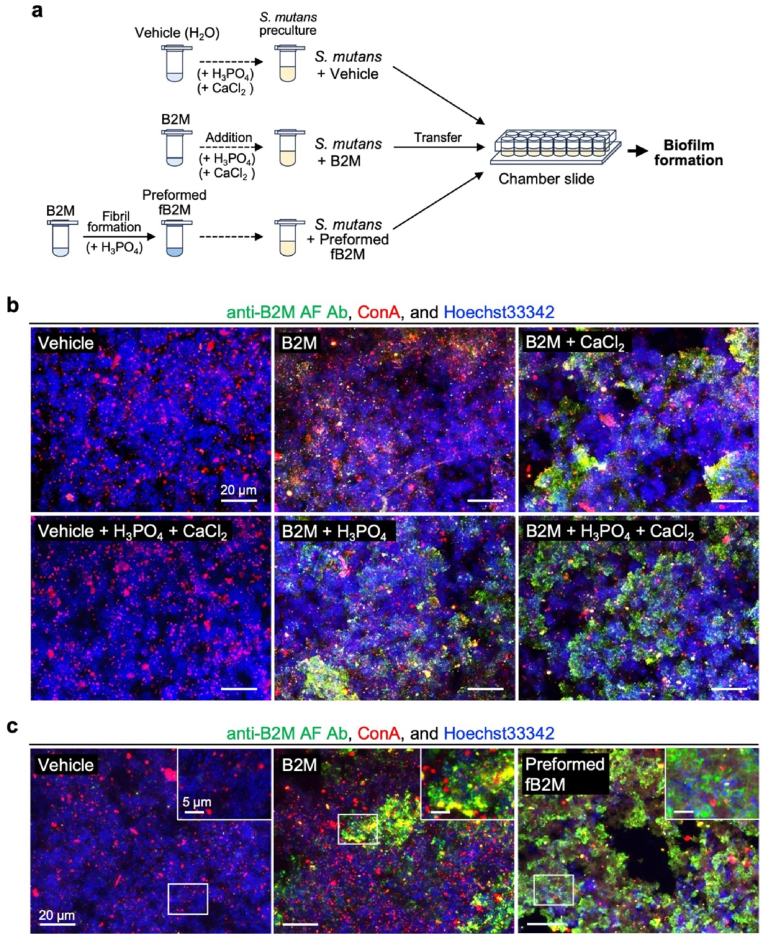
*Streptococcus mutans* biofilm induces β_2_-microglobulin (B2M) fibrillation, incorporating the resulting fibrils into the biofilm. (a) The experimental scheme of *S. mutans* biofilm formation with vehicle, B2M, and fibrillar B2M (fB2M). **(b)** Confocal microscopy images of *S. mutans* biofilms formed in the presence of B2M with anions and/or divalent cations abundant in the oral environment and were compared to those formed in the absence of B2M (vehicle control; H_2_O). *S. mutans* was incubated at 37 °C for 8 h in brain heart infusion (BHI) broth supplemented with 1 % sucrose, in the presence of B2M with 2.5 mM H_3_PO_4_ and/or 5 mM CaCl_2_. Biofilms were subjected to triple fluorescence staining with an anti-B2M AF Ab (green), ConA (red, indicating extracellular glycoconjugates), and Hoechst 33342 (blue, for *S. mutans* visualization). Scale bars: 20 μm. **(c)** Confocal microscopy images of *S. mutans* biofilms formed in the presence of preformed B2M. *S. mutans* was incubated at 37 °C for 8 h in BHI broth supplemented with 1 % sucrose, in the presence of 2.5 mM H_3_PO_4_ (vehicle control), B2M (100 μg/mL in 2.5 mM H_3_PO_4_), or preformed fB2M (100 μg/mL formed in 2.5 mM H_3_PO_4_). Biofilms were subjected to triple fluorescence staining with an anti-B2M AF Ab (green), ConA (red, indicating extracellular glycoconjugates), and Hoechst 33342 (blue, for *S. mutans* visualization). Scale bars: 20 μm. Inset images show magnified views of the areas outlined in the white line. Scale bars: 5 μm. (For interpretation of the references to colour in this figure legend, the reader is referred to the Web version of this article.)

To assess the interaction between B2M and dental plaque biofilms, we utilized a *S. mutans* biofilm model (Fig. 4a). During 8 h of biofilm formation, the medium pH decreased from 6.2 to 4.7 (Fig. S5a), and only minimal fB2M dissociation occurred under these mildly acidic conditions (Fig. S5b and c). When *S. mutans* was cultured with native B2M alone, fB2Ms were scarcely formed in the biofilm (Fig. 4b); however, in the presence of H_3_PO_4_ or CaCl_2_, the fB2Ms were incorporated into the biofilm and appeared locally abundant. Such local presence of fB2M in this model is similar to the fB2M localization observed in dental plaque specimens. Intriguingly, in the presence of H_3_PO_4_ and CaCl_2_, fB2Ms were incorporated throughout the biofilm (Fig. 4b), where the amount of fB2M incorporated varied, with certain regions exhibiting abundant fB2M accumulation. Thus, native B2M transforms into fB2M in the presence of the plaque environmental factors PO_4_^3−^ and Ca^2+^, and is incorporated into biofilms.

Next, we investigated how fB2M could be incorporated into the biofilm. To this end, we added artificially fibrillized B2M (preformed fB2M) to the *S. mutans* biofilms (Fig. 4a) and found that the preformed fB2Ms can be extensively incorporated throughout the biofilm, in contrast to the partial incorporation of native B2M (Fig. 4c). This observation suggests that the entire biofilm has the property of incorporating exogenous fB2Ms; however, the property of converting B2M into fibrils is possessed only locally.

These findings demonstrate that Ca^2+^ and PO_4_^3−^-rich environments, which form locally on biofilm surfaces, convert native B2M into fB2M that is subsequently incorporated into the biofilm.

### 2.5 B2M loses antimicrobial activity through fibril formation and enhances biofilm growth

**Fig. 5:**
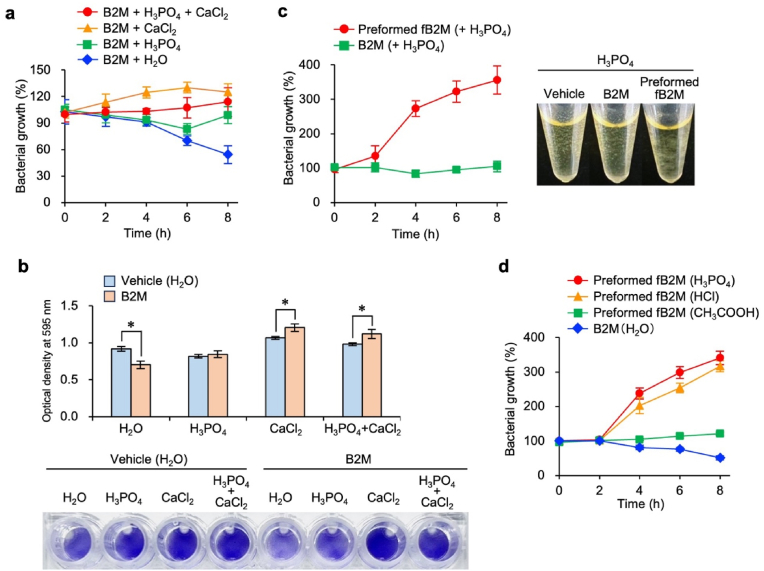
β_2_-microglobulin (B2M) loses antimicrobial activity through fibrillar B2M (fB2M) formation and promotes biofilm formation. (a) Effects of B2M on *S. mutans* growth in the presence of H_3_PO_4_ and/or CaCl_2_. Bacterial growth (%) was assessed in the presence of B2M (100 μg/mL) under indicated conditions (H_2_O, 2.5 mM H_3_PO_4_, 5 mM CaCl_2_, or their combination) and normalized to respective vehicle controls without B2M at each time point (0−8 h). **(b)** Effects of B2M on *S. mutans* biofilm formation under the same conditions, as shown in (a). Bacterial mass in the adherent biofilms was quantified at 595 nm (upper panel). Stained adherent biofilms of *S. mutans* formed in each culture are shown in the picture (lower panel). Data are expressed as the mean ± SD (n ≥ 3) based on at least three independent experiments. ∗*P* < 0.01 compared with the respective vehicle control. **(c)** Effect of preformed fB2M on the growth of *S. mutans* (left). *S. mutans* growth was assessed in the presence of either 2.5 mM H_3_PO_4_ (vehicle control), B2M (100 μg/mL in 2.5 mM H_3_PO_4_), or preformed fB2M (100 μg/mL in 2.5 mM H_3_PO_4_). Bacterial growth (%) was normalized to that of the vehicle control at each time point (0−8 h). Representative culture images used to measure bacterial growth show increased biofilm formation in the tube of the sample with preformed fB2M (right). **(d)** Effect of fB2M preformed under different acidic conditions on *S. mutans* growth. The growth of *S. mutans* was assessed in the presence of B2M (100 μg/mL in H_2_O) or preformed fB2M (100 μg/mL) in 2.5 mM H_3_PO_4_, 2.5 mM HCl, or 25 mM CH_3_COOH. Bacterial growth (%) was normalized to that of the respective vehicle control (H_2_O instead of B2M and preformed fB2M in each solvent) at each time point (0−8 h).

We subsequently examined the influence of native B2M or fB2Ms on the growth of *S. mutans* biofilms. The growth of *S. mutans*, assessed via ATP production and biofilm formation, was significantly suppressed in the presence of native B2M (Fig. 5a and b), indicating that native B2M exerts antimicrobial activity. In contrast, under fB2M-forming conditions with H_3_PO_4_, CaCl_2_, or their combination, B2M no longer suppressed *S. mutans* growth or biofilm formation; instead, it slightly promoted both (Fig. 5a and b). These results suggest that B2M loses its antimicrobial activity through fibril formation and fB2Ms promote biofilm growth.

We therefore investigated the biofilm growth-promoting effect of preformed fB2Ms with the property of being incorporated throughout the *S. mutans* biofilm (Fig. 4c). Notably, the preformed fB2Ms increased *S. mutans* growth by approximately threefold (Fig. 5c). Furthermore, we examined whether there were any differences among fB2Ms formed in different acids. The fB2Ms formed in the presence of HCl or H_3_PO_4_—which generate typical elongated fibrils (Fig. 3)—exhibited an obvious growth-promoting effect (Fig. 5d). In contrast, the fB2Ms formed in the presence of CH_3_COOH—which generate atypical fibrils (Fig. 3)—showed no growth-promoting effect (Fig. 5d). These findings suggest that the shape of fB2Ms can affect biofilm development.

### 2.6 fB2M incorporation alters the structural properties of the biofilm matrix and reduces biofilm adhesion

**Fig. 6:**
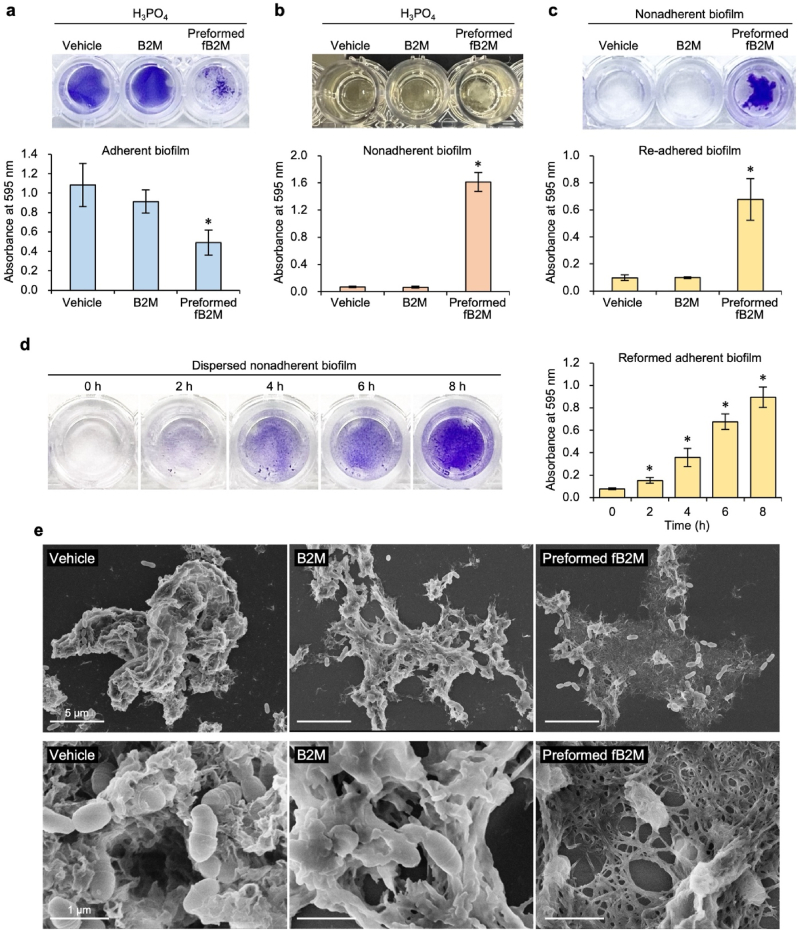
Incorporation of large amounts of β_2_-microglobulin (B2M) fibril alters biofilm adhesiveness and matrix architecture. (a) Effects of preformed fibrillar B2M (fB2M) on the amount of *S. mutans* biofilms. *S. mutans* was incubated in brain heart infusion (BHI) broth supplemented with 1 % sucrose at 37 °C for 8 h, in the presence of either 2.5 mM H_3_PO_4_ (vehicle control), B2M (100 μg/mL in 2.5 mM H_3_PO_4_), or preformed fB2M (100 μg/mL in 2.5 mM H_3_PO_4_). Representative pictures of adherent biofilms stained with crystal violet are shown (upper panel). The bacterial mass in adherent biofilms was quantified based on the absorbance of the destaining solution at 595 nm (lower panel). Data represent at least three independent experiments and are presented as the mean ± SD (n ≥ 3). ∗*P* < 0.01 compared to vehicle or B2M. **(b)** Representative pictures of *S. mutans* cultures (upper). Nonadherent biofilms were observed only in the presence of preformed fB2M. Nonadherent biofilms in the culture supernatants were collected through centrifugation and stained with 0.1 % crystal violet. The biofilm biomass was quantified by measuring the absorbance of the destaining solution at 595 nm (lower). Data are expressed as the mean ± SD (n ≥ 3) based on at least three independent experiments. ∗*P* < 0.001 compared to all other groups. **(c)** Re-adhesion capacity of nonadherent *S. mutans* biofilms to new culture vessels. *S. mutans* biofilms were formed in the presence of either 2.5 mM H_3_PO_4_ (vehicle control), B2M (100 μg/mL in 2.5 mM H_3_PO_4_), or preformed fB2M (100 μg/mL in 2.5 mM H_3_PO_4_). Nonadherent biofilms in supernatants were collected, resuspended in BHI broth supplemented with 1 % sucrose, and used to assess re-adhesion capacity. Representative images of reformed adherent biofilms stained with crystal violet are shown (upper panel). The bacterial mass in reformed adherent biofilms was quantified based on the absorbance of the destaining solution at 595 nm (lower panel). Data represent at least three independent experiments and are presented as the mean ± SD (n ≥ 3). ∗*P* < 0.01 compared to all other groups. **(d)** Time-course of biofilm reformation by dispersed nonadherent *S. mutans* biofilms. Nonadherent biofilms were collected as described in (c), diluted 10-fold in BHI broth supplemented with 1 % sucrose, dispersed by vigorous pipetting, and used for reformation assays. Representative images of reformed adherent biofilms stained with crystal violet (left panel). The bacterial mass in reformed adherent biofilms was quantified based on the absorbance of the destaining solution at 595 nm (right panel). Data represent at least three independent experiments and are presented as the mean ± SD (n ≥ 3). ∗*P* < 0.05 compared to the previous time point. **(e)** Scanning electron microscopy (SEM) images of *S. mutans* biofilms formed in the presence of B2M or preformed fB2M. *S. mutans* was cultured at 37 °C for 4 h in BHI broth supplemented with 1 % sucrose in the presence of either 2.5 mM H_3_PO_4_ (vehicle control), B2M (100 μg/mL in 2.5 mM H_3_PO_4_), or preformed fB2M (100 μg/mL in 2.5 mM H_3_PO_4_). Each SEM image was captured at magnifications of × 5000 (upper) and × 30,000 (lower). Scale bars: 5 μm and 1 μm, respectively. (For interpretation of the references to colour in this figure legend, the reader is referred to the Web version of this article.)

Next, we examined the impact of fB2Ms on the adhesive property of *S. mutans* biofilms. fB2Ms generated under phosphate-rich conditions significantly enhanced biofilm formation (Fig. 5a and b). Paradoxically, quantitative analysis revealed a marked decrease in surface-adherent biofilms within the culture vessels (Fig. 6a), with a substantial increase in nonadherent biofilm biomass (Fig. 6b). These nonadherent biofilms re-adhered to new culture vessels (Fig. 6c). After dispersion by vigorous pipetting, they gradually reformed adhesive biofilms during subsequent culturing (Fig. 6d). Scanning electron microscopy (SEM) images demonstrated distinct structural differences among biofilms: native B2M-added biofilms maintained bacterial density and extracellular matrix volume comparable to the controls, whereas preformed fB2Ms induced matrix-dominated biofilms featuring a reticulated architecture enveloping bacterial cells (Fig. 6e). This structural reorganization contrasts sharply with the homogeneous membrane-like matrix observed in native B2M biofilms.

These findings demonstrate that biofilm-incorporated fB2Ms modulate critical biofilm characteristics, including biomass, structural organization of the matrix, and adhesive capacity.

## 3 Discussion

To the best of our knowledge, this study is the first to demonstrate that dental plaque biofilms interact with B2M, a prevalent salivary antimicrobial protein, converting it into fibrils. This process disables its antimicrobial function and integrates it into the plaque matrix, potentially affecting biofilm growth and adhesion. This mechanism suggests that dental plaque biofilms employ a specialized strategy to neutralize host defense factors, thereby facilitating their persistence. Additionally, host-derived exogenous fibrils may act as structural elements aiding biofilm growth, revealing complex interactions between host factors and bacterial communities in dental plaque. Furthermore, we successfully visualized detailed fibril structures in plaque specimens using immunofluorescence microscopy for the first time. While prior studies inferred bacterial AF presence using Congo red birefringence and ThT fluorescence [[35], [36], [37]], detailed structural visualization has remained lacking. Therefore, our findings provide novel insights into dental plaque biology and underscore the vital role of host-derived fibrils in biofilm formation.

The role of host-derived exogenous proteins in fibril formation and their subsequent accumulation within dental plaque biofilms has not been explored. Previous research has suggested that several oral streptococci, such as *S. mutans* and *Streptococcus sanguinis*, produce bacterial AFs as structural components of biofilms [[35], [36], [37]]. In our study, we identified the presence of B2M-derived fibrils by immunofluorescence using the anti-B2M AF Ab in dental plaque specimens. Additionally, using the anti-IAPP AF Ab, general fibril structures were ubiquitously found across the specimens, possibly originating from both host and bacterial sources. Prior studies have reported that oral streptococci require 3–5 days to produce detectable levels of bacterial AFs [36,37]. In contrast, our dental plaque specimens were collected just 1–2 days after tooth brushing, suggesting that the detected fibrils form earlier than bacterial AFs and can primarily result from the fibrillation of host-derived proteins such as B2M. This temporal discrepancy suggests that host-derived exogenous proteins, such as B2M, are the primary source of early-stage fibril formation, potentially providing beneficial structural scaffolds for initial biofilm growth. Bacterial AFs contribute to biofilm formation, structural integrity, and robustness through interactions with extracellular DNA and polysaccharides [[38], [39], [40], [41], [42]]. Host-derived fibrils like fB2Ms may also participate in early biofilm development through interactions with these matrix components. Additionally, bacterial amyloidogenic proteins can induce fibril formation in other bacterial species and in host proteins through cross-seeding, in which amyloid nuclei from one species promote aggregation in another species, thereby supporting the structure of multi-species biofilms [[43], [44], [45]]. Thus, fB2Ms formed through cross-seeding with bacterial amyloidogenic proteins may further reinforce biofilm development and stability.

We observed variations in the detection levels of B2M-derived fibrils across the dental plaque specimens. These differences likely arise from multiple factors, including salivary B2M concentrations, the availability of PO_4_^3−^ and Ca^2+^—which are critical for fB2M formation—fluctuations in dental plaque pH, and the prevalence of SCFA-producing bacteria. The acidic conditions required for B2M fibrillation can be induced by distinct bacterial species that produce varying SCFA profiles, creating diverse microenvironmental conditions within the plaque [46]. Furthermore, additional factors, such as the inflammatory state and the presence of pathogenic bacteria, such as *Porphyromonas gingivalis*, may influence B2M stability in dental plaque [47], thereby affecting the extent of fibril formation. Further studies remain warranted to elucidate the impact of these factors on fibril formation and the dynamics of host-derived proteins.

We demonstrated that B2M forms diverse structures depending on the type and concentration of coexisting anions (Cl^−^, PO_4_^3−^, or SCFA anions) (Fig. 3). These morphological differences may reflect anion lyotropic effects, as stronger lyotropic anions in the Hofmeister series enhance protein hydrophobic interactions by modifying water-protein interfaces [48,49]. The anion ranking in the Hofmeister series is PO_4_^3−^ > CH_3_COO^−^ > Cl^−^, indicating differential impacts on B2M's intra- and intermolecular hydrophobic interactions, leading to diverse fibril morphologies in this study. ThT fluorescence intensity was higher under 100 mM SCFA conditions than under 10 mM HCl or H_3_PO_4_, likely attributable to the quantity and structural properties of the shorter, thicker fibrillar structures observed under 100 mM SCFA (Fig. 3). Acidity-induced structural denaturation, salt-driven alterations, and anion-binding effects likely synergize in fB2M formation [30,50]. Furthermore, the presence of Cl^−^ and PO_4_^3−^ with Ca^2+^ or Mg^2+^ enhanced fB2M formation, yielding bundled fibrillar structures under acidic conditions. Prior studies suggest that Ca^2+^, Mg^2+^, Cu^2+^, and Zn^2+^, among other divalent cations, promote B2M insolubilization and mesh-like fibril formation under physiological conditions, probably through divalent cation binding to calcium-binding motifs (DXD/DXE/EXD) located near the loop structures between two β-sheets of B2M [32,51,52]. Given that Ca^2+^ increases the binding affinity of aggregated B2M to *S. mutans* surfaces [22], the observed enhancement is likely induced by such Ca^2+^-mediated fibril formation. Thus, the presence of PO_4_^3−^ and SCFA-derived anions, combined with divalent cations such as Ca^2+^ and Mg^2+^ in the dental plaque environment, likely triggers structural changes in B2M, facilitating fibril formation and morphological diversity.

Although physical stimuli are typically deemed essential for B2M AF formation [28,31], the specific triggers in dental plaque remain to be determined. B2M formed fibrils in an *S. mutans* biofilm model without external physical stimuli, indicating that the biofilm microenvironment itself fostered B2M fibrillation. During *S. mutans* biofilm formation, we found that B2M underwent fibrillation and was integrated into the biofilm matrix. While native monomeric B2M showed antimicrobial activity, this was lost upon fibril formation. Notably, fB2M incorporation enhanced biofilm growth, possibly serving as a structural scaffold within the matrix. Similar to bacterial endogenous AFs in *S. mutans* biofilms that aid structural stabilization and development [53], host-derived fB2Ms may have comparable effects on biofilm matrix architecture. The mechanism behind fB2M promotion of *S. mutans* growth remains to be elucidated.

While supporting bacterial growth, fB2Ms also alter the physical properties of biofilms. Our experiments with preformed fB2Ms showed a significant reduction in biofilm adhesive properties. SEM observations revealed that fB2Ms reshaped the biofilm matrix into a reticulated structure, likely altering biofilm adhesion. During *S. mutans* biofilm maturation, bacterial SCFAs may trigger B2M conformational changes, leading to localized fibril formation near bacterial cells. Overall fibril content in the biofilm may remain low, limiting its effect on adhesion, but regions with high fibril concentrations were observed, suggesting spatially variable reductions in adhesive capacity. Consistently, the *S. mutans*-derived amyloidogenic protein adhesin P1 reduces biofilm adhesion when forming AFs during late-stage biofilm growth [36,54]. We further demonstrated that nonadherent biofilms possess the ability to reattach and reform adherent biofilms. This suggests that the formation of nonadherent biofilm fragments by fB2M may serve as a mechanism for spatial expansion of colonization within the oral cavity. This dispersal–reformation cycle may also contribute to the persistence of dental plaque.

**Fig. 7:**
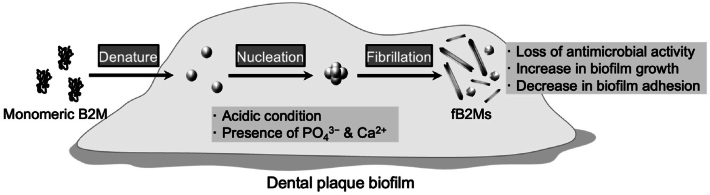
Schematic diagram of β_2_-microglobulin (B2**M) functional transformation through fibrillation in the dental plaque environment.** Salivary monomeric B2M encounters dental plaque biofilms in acidic microenvironments rich in phosphate and calcium ions. Under these conditions, B2M undergoes conformational changes, triggering nucleation. These nuclei rapidly polymerize and assemble into mature fibrillar B2Ms (fB2Ms). This transformation converts B2M, an antimicrobial protein, to a structural component of biofilm, paradoxically reducing surface adhesion while facilitating bacterial dissemination and oral expansion.

In Fig. 7, we propose that B2M fibrillation in dental plaque biofilm environments serves as a “molecular hijacking” strategy, enabling biofilms to neutralize host antimicrobial factors and achieve structural integrity. Salivary monomeric B2M encounters acidic microenvironments in dental plaque rich in PO_4_^3−^ and Ca^2+^, triggering conformational changes and nucleation—the rate-limiting step in fibril formation. These nuclei rapidly polymerize into mature fB2Ms with or without cross-β sheet architecture. This transformation alters the function, as it loses its antimicrobial activity while gaining properties that enhance biofilm growth. Paradoxically, these fibrils reduce biofilm adhesion to tooth surfaces, potentially facilitating bacterial dissemination and oral biofilm expansion, which may contribute to the pathogenicity of dental plaque. Future studies should investigate other host antimicrobial proteins, such as cystatin C, lactoferrin, lysozyme, and S100A8/A9, for their role in fibril formation within dental plaque microenvironments.

In conclusion, we identified a novel adaptive mechanism by which dental plaque biofilms convert B2M, an exogenous host antimicrobial protein, into fibrils, neutralizing its antimicrobial activity and incorporating the resulting fibrils as structural components of the biofilm. We hypothesize that dental plaque employs specialized strategies to counteract host defense mechanisms and ensure its persistence. Future studies should explore the dynamics between dental plaque biofilms and diverse antimicrobial agents, as well as the spatiotemporal regulation of biofilm growth under these conditions. This will enhance our understanding of host-microorganism interactions and facilitate the development of innovative plaque control strategies, including inhibitors or modulators to prevent amyloidogenesis within biofilm microenvironments.

## 4 Materials and methods

### 4.1 Reagents and Abs

Native human B2M protein, purified from human urine, was obtained commercially as a lyophilized form after being freeze-dried from a B2M solution in 20 mM ammonium bicarbonate buffer (a volatile buffer). For experimental use, the B2M powder was reconstituted in Milli-Q ultrapure water and stored at −80 °C until use. The human IAPP (Cat# 4219-v) and amyloid b (1-42) peptide (Aβ; Cat# 4349-v) were obtained from Peptide Institute. ThT (Cat# 202–01002) was purchased from FUJIFILM Wako. The anti-B2M monoclonal Ab (clone EP2978Y, Cat# ab75853) was obtained from Abcam. Sigma-Aldrich provided anti-AF LOC Ab (Cat# AB2287), a rabbit polyclonal Ab raised against AFs derived from IAPP, which specifically recognizes generic epitopes common in several AFs and fibrillar oligomers, but not in monomers, prefibrillar oligomers, or natively folded proteins. Other reagents were obtained from FUJIFILM Wako.

### 4.2 Collection of dental plaque specimens

Supragingival dental plaque specimens were collected from 14 healthy human participants aged 16–56 years (Table S3), following a previously described protocol [11]. Specimens were designated using a hash symbol (#) followed by a unique identification number (e.g., #1). The alphabetical designations of the specimens shown in Fig. 1b correspond to the specimen numbers a (#5), b (#4), c (#3), d (#2), and e (#1). The participants had no severe health conditions such as diabetes, hypertension, or infectious diseases, and were not taking any medication. The study was approved by the Ethics Committee of our University (approval number 34022); informed consent was obtained from participants, or their parents or guardians (if age <18 years). Before the specimen collection, participants were visually examined to confirm the absence of inflammatory signs. The supragingival plaque specimens were carefully collected from the molar region of the upper or lower jaw using a sterile dental probe, immediately suspended in ice-cold phosphate-buffered saline (PBS) supplemented with cOmplete EDTA-free Protease Inhibitor Cocktail Tablets (Roche, Cat# 4693132001), and stored at −80 °C until further analysis. Protein concentrations were determined using a Pierce BCA Protein Assay Kit (Thermo Fisher Scientific, Cat# 23227). Each dental plaque suspension (50 μL) was fractionated by centrifugation at 15,000×*g* for 10 min at 4 °C. Supernatants (soluble fractions) were transferred to new 1.5 mL microtubes, while pellets (insoluble fractions) were resuspended in 50 μL of PBS.

### 4.3 In vitro fibril formation

To investigate the effect of HCl or H_3_PO_4_ on fB2M formation, 50 μL of B2M (0.5 mg/mL [42 μM]) was incubated in 1 or 10 mM HCl or H_3_PO_4_ in the presence of 150 mM NaCl for 0−24 h at 37 °C. The pH values of the reaction mixtures with 1 mM HCl and H_3_PO_4_ were 2.7 and 2.8, respectively, while those with 10 mM HCl and H_3_PO_4_ were 2.0 and 2.1, respectively. To examine the impact of bacterial SCFAs on fB2M formation, 50 μL of B2M (0.5 mg/mL) was incubated in 100 mM CH_3_COOH, CH_3_(CH_2_)_2_COOH, CH_3_CH(OH)COOH, or CH_3_CH_2_COOH in the presence of 150 mM NaCl at 37 °C for 0−24 h. The pH values of the reaction mixtures with 100 mM solutions of CH_3_COOH, CH_3_(CH_2_)_2_COOH, CH_3_CH(OH)COOH, and CH_3_CH_2_COOH were 2.8, 2.7, 2.1, and 2.6, respectively. The effect of divalent cations (Ca^2+^ and Mg^2+^), which are present in dental plaque, on fB2M formation was assessed by incubating 50 μL of B2M (0.5 mg/mL) in 10 mM H_3_PO_4_ or 100 mM CH_3_COOH containing either 150 mM CaCl_2_, MgCl_2_, NaCl, or 300 mM NaCl at 37 °C for 0−24 h. During incubation, all samples used in these assays were inverted intermittently at 10 rpm. The formation of fB2M under each reaction condition was assessed by measuring ThT fluorescence intensity.

IAPP-derived and Aβ-derived fibrils were prepared by incubating 50 μL of IAPP (100 μg/mL) and Ab (100 μg/mL), respectively, in PBS under static conditions at 37 °C for 8 h, with continuous tumbling at 10 rpm. Fibril formation was confirmed based on ThT fluorescence intensity. These fibrils were used as positive controls and comparative standards in dot blot analyses, as well as to validate the specificity of the anti-B2M AF Ab.

### 4.4 Generation of polyclonal Ab against fB2M

A mouse anti-B2M AF polyclonal Ab was generated in 5–8-week-old female BALB/c mice through five rounds of immunization, following a previously described protocol [11]. fB2M was prepared by incubating native human B2M in 10 mM HCl with 150 mM NaCl at 37 °C for 16 h, with continuous tumbling at 10 rpm using an established method for B2M amyloidogenesis [28]. For immunization, an initial subcutaneous injection of 250 μg fB2M emulsified with complete Freund's adjuvant on day 0 was followed by four booster injections of the same dose of antigen emulsified with incomplete Freund's adjuvant on days 14, 28, 42, and 56. Serum samples were collected on day 70 after the initial immunization. All the above procedures were outsourced to Biogate (Yamagata, Japan).

Abs titers and specificities were evaluated using an indirect enzyme-linked immunosorbent assay (ELISA). For comparative immunoreactivity assay, MaxiSorp immunoassay plates (Nunc, Cat# 439454) were coated with 100 μL/well of fB2M (0.1 μg) and incubated overnight at 4 °C. After three washes with PBS supplemented with 0.1 % Tween 20 (PBS-T), plates were blocked using 1 % bovine serum albumin (BSA; 200 μL/well) at room temperature (RT) for 1 h. After three additional washes with PBS-T, plates were incubated with 100 μL of 10-fold serially diluted anti-B2M AF polyclonal Ab at RT for 2 h. The pre-immune serum served as a negative control in our specificity assessment. To validate cross-reactivity, MaxiSorp immunoassay plates were coated with 100 μL/well of 10-fold serially diluted fB2M, IAPP fibril, or Aβ fibril and incubated overnight at 4 °C. After three washes with PBS-T, plates were blocked using 1 % BSA (200 μL/well) at RT for 1 h. After three additional washes with PBS-T, plates were incubated with 100 μL of diluted anti-B2M AF polyclonal Ab (1:10,000) at RT for 2 h. Subsequently, plates were washed three times using PBS-T and incubated with horseradish peroxidase (HRP)-conjugated goat anti-mouse IgG (1:20,000 dilution; Medical & Biological Laboratories, Cat# 330) at RT for 1 h. The chromogenic reaction was initiated by adding 100 μL of TMB Solution (Beacle, Cat# BCL-TMB-01) per well and allowed to develop at RT for 15 min. The reaction was terminated by adding 1 N sulfuric acid, and the absorbance at 450 nm was measured using an Infinite M Plex multimode plate reader.

### 4.5 Immunofluorescence staining on dental plaque specimens

Dental plaque specimens (n = 5) were evenly smeared onto APS-coated glass slides, fixed by incubating with 4 % PFA at RT for 10 min, and blocked with Blocking One Histo blocking reagent at RT for 10 min. For immunostaining, samples were incubated with a rabbit anti-AF LOC polyclonal Ab at RT for 60 min, followed by Alexa 488-conjugated anti-rabbit IgG (Thermo Fisher Scientific, Cat# A11034) at RT for 30 min to visualize fibrils. fB2Ms were labeled with mouse polyclonal serum against fB2M serving as the primary Ab at RT for 60 min, followed by incubation with Alexa 488-conjugated anti-mouse IgG (Thermo Fisher Scientific, Cat# A11029) used as the secondary Ab at RT for 30 min. The specimens were visualized using Alexa 594-conjugated concanavalin A (ConA) at RT for 30 min, according to the manufacturer's instructions. Between each incubation step, the slides were washed three times with PBS-T. All immunofluorescence assays were performed in the dark.

Immunolabeled specimens were mounted using the ProLong Glass Antifade Mountant. Fluorescence imaging was performed using a Zeiss LSM 710 confocal laser-scanning microscope (Carl Zeiss) equipped with the following filter sets: green fluorescence (excitation, 488 nm; dichroic mirror, MBS 488/561; emission, 509 nm), and red fluorescence (excitation, 568 nm; dichroic mirror, MBS 488/561; emission, 602 nm). For each sample, Z-stack acquisition was performed for approximately 30 min. Images acquired using a × 63 oil immersion objective lens (numerical aperture, 1.4) were digitally processed at a final magnification of × 630. These imaging parameters were consistently used throughout the study. For each of the five specimens, at least 10 fields of view were imaged, and representative images were presented.

A series of control experiments was performed to validate the specificity of our immunostaining protocol. The autofluorescence of unstained dental plaque specimens was assessed to establish baseline signal levels. Secondary Ab controls, without primary Ab reaction, were used to evaluate non-specific binding. Specificity of the primary Abs was verified using an isotype-matched control Ab (IgG1κ), which revealed no detectable signal. Pre-immune mouse serum served as a negative control for the anti-B2M AF polyclonal Ab, resulting in no detectable fluorescence. These controls collectively ensured the internal validity of the immunostaining results and minimized the risk of false-positive signals.

### 4.6 Detection of fibrils by ThT fluorescence

ThT is a benzothiazole dye that emits strong fluorescence at ∼482 nm upon excitation at ∼450 nm when bound to fibrils. An aliquot (5 μL) of the sample of *in vitro* fibril formation was mixed with 95 μL of 20 μM ThT solution in 100 mM sodium phosphate buffer (pH 7.4). The fluorescence intensity of ThT bound to fibrils was measured using an Infinite M Plex multimode plate reader (Tecan), with excitation at 445 nm and emission at 485 nm. ThT fluorescence intensities detected through the *in vitro* fibril formation assay were expressed as relative fluorescence units (RFU) after subtracting the background fluorescence intensity of ThT alone in 100 mM sodium phosphate buffer (pH 7.4). ThT fluorescence intensity was evaluated using at least three biological replicates.

### 4.7 Dot blotting

The presence of AFs and/or fB2Ms in diluted dental plaque specimens was assessed using dot blotting, in which Amersham polyvinylidene difluoride (PVDF) membranes (Cytiva, Cat# 10600023) were cut, incubated in methanol at RT for 3 min, and equilibrated in PBS-T at RT for 30 min. Diluted dental plaque specimens (100 μL) were applied to the membranes, followed by rapid immobilization using a Bio-Dot microfiltration apparatus (Bio-Rad Laboratories). After washing with PBS-T, membranes were blocked using EzBlock Chemi blocking solution (ATTO, Cat# AE-1475) at RT for 30 min. After three washes with PBS-T, the membranes were incubated with mouse anti-B2M AF polyclonal Ab (1:10,000) or rabbit anti-AF LOC polyclonal Ab (1:5000) at RT for 60 min. Next, the membranes were washed with PBS-T three times for 10 min each at RT, followed by incubation with HRP-conjugated secondary Ab (either goat anti-mouse IgG [1:20,000] or goat anti-rabbit IgG [1:10,000]; Medical & Biological Laboratories, Cat# 458) at RT for 30 min.

Both the soluble and insoluble fractions of the dental plaque specimens (10 μL) were rapidly immobilized onto the membranes and then blocked as described above. After three washes with PBS-T, the membranes were incubated at RT for 60 min with any of the rabbit anti-AF LOC polyclonal Ab (1:5000), rabbit anti-B2M monoclonal Ab (1:10,000), and mouse anti-B2M AF polyclonal Ab (1:10,000). Membranes were then incubated at RT for 30 min with the appropriate HRP-conjugated secondary Ab (either goat anti-rabbit IgG [1:10,000 or 1:20,000] or goat anti-mouse IgG [1:20,000]).

After three washes with PBS-T, chemiluminescent signals on each PVDF membrane were detected using Amersham ECL Select (Cytiva, RPN2235) and visualized using a LuminoGraph III chemiluminescent imaging system (ATTO).

### 4.8 Western blotting

The soluble and insoluble fractions of the dental plaque specimens (20 μL) were subjected to sodium dodecyl sulfate-polyacrylamide gel electrophoresis using a 4–20 % gradient polyacrylamide gel (ATTO, Cat# 2331304) and transferred to PVDF membranes. Membranes were blocked using EzBlock Chemi blocking solution at RT for 30 min. After three washes with PBS-T, the membranes were incubated at RT for 60 min with rabbit monoclonal anti-B2M Ab (1:5000). The membranes were then incubated at RT for 30 min with HRP-conjugated goat anti-rabbit IgG (1:10,000). After three washes with PBS-T, the chemiluminescent substrate Amersham ECL Select was used to develop the signals from each PVDF membrane. The signals were visualized using a LuminoGraph III chemiluminescent imaging system.

### 4.9 TEM

Aliquots (5 μL) of each B2M sample after treatment were deposited onto a carbon-coated copper grid (Excel support film, Nissin EM, Cat# 649) and incubated at RT for 5 min. For negative staining, samples were treated with 10 μL of 2 % uranyl acetate at RT for 5 min. Excess liquid was carefully removed using filter paper, followed by a rapid rinsing with Milli-Q ultrapure water. The grids were air-dried, and TEM was performed using a JEM-3010 electron microscope (JEOL) operated at an accelerating voltage of 100 kV. Micrographs were acquired at magnifications of×2,000, × 15,000, × 25,000, and × 60,000 using the same imaging parameters throughout the study. For each sample, 10 fields of view were recorded in at least three independent experiments, and representative images were selected for figure presentation.

### 4.10 Bacterial strain and growth conditions

*S. mutans* ATCC 25175 was used as a model biofilm-forming oral bacterial species. The bacterial strain was aerobically cultured in modified brain heart infusion (BHI) broth (Becton Dickinson, Cat# 237500) and on BHI agar plates, with or without 1 % (w/v) sucrose, at 37 °C for 12–16 h. The bacterial growth kinetics were spectrophotometrically analyzed by measuring the optical density at 600 nm (OD_600_), which was calibrated to known cell numbers, and used to estimate the cell density. Bacterial cells in the logarithmic growth phase were harvested by centrifugation at 3000×*g* at RT for 5 min and used for the experiments described below.

### 4.11 pH monitoring during S. mutans biofilm formation

*S. mutans* was aerobically cultured at 37 °C in BHI broth until the mid-logarithmic growth phase (OD_600_ ≈ 1.0) was reached. To monitor pH changes during biofilm formation, the culture was diluted 1:60 in BHI broth supplemented with 1 % (w/v) sucrose. Aliquots of 180 μL from the bacterial suspension were distributed into individual wells of a flat-glass-bottomed 16-well Lab-Tek Chamber Slide System (Nunc, Cat# 178599). The bacterial cultures were treated with vehicle (H_2_O) or 10 mM H_3_PO_4_ (each 60 μL) in the presence of 150 mM NaCl at 37 °C under aerobic conditions for 0, 2, 4, 6, or 8 h. At each time point, the culture medium from each well was collected into 1.5-mL tubes and centrifuged at 15,000×*g* and RT for 2 min. The bacteria-free culture medium (200 μL) was then collected, and the pH was measured using a portable pocket pH meter (LAQUAtwin pH, Horiba, Cat# pH-33B).

### 4.12 Assessment of fB2M stability at different pH values

To evaluate the stability of fB2Ms at different pH levels, 50 μL of B2M (0.5 mg/mL [42 μM]) was incubated in 10 mM H_3_PO_4_ with 150 mM NaCl for 16 h at 37 °C under continuous tumbling at 10 rpm to induce fibril formation. The resulting fB2Ms were collected at the bottom of 1.5-mL tubes by centrifugation at 15,000×*g* and RT for 10 min, and the supernatant was carefully removed.

The sedimented fB2Ms were washed twice with 100 μL of 10 mM or 10 μM phosphoric acid (pH 2.0 or pH 4.7), 20 mM sodium phosphate buffer (pH 2.0, pH 6.2, or pH 7.4), or 20 mM sodium citrate buffer (pH 4.7), each containing 150 mM NaCl. After washing, the fibrils were resuspended in 50 μL of the corresponding buffer solution. Each fB2M suspension was incubated at 37 °C for 0, 2, 4, 6, or 8 h. To assess fB2M stability, the fluorescence intensity of ThT bound to the fB2Ms was measured using an Infinite M Plex multimode plate reader (Tecan), with excitation at 445 nm and emission at 485 nm. The resulting ThT fluorescence intensities were expressed as the relative ThT fluorescence ratio, calculated as the percentage of the RFUs of fB2Ms at 0 h for each pH condition. Each experiment was performed with at least three biological replicates.

### 4.13 S. mutans biofilm formation

*S. mutans* was aerobically cultured at 37 °C in BHI broth until the mid-logarithmic phase was reached. For the biofilm formation assay, the overnight culture was diluted 1:60 in BHI broth supplemented with 1 % (w/v) sucrose. Aliquots (120 μL) of the bacterial suspension were inoculated into individual wells of a flat-glass-bottomed 16-well Lab-Tek Chamber Slide System. The wells were incubated with vehicle (H_2_O), native B2M, or preformed fB2M (each 40 μL; final concentration, 100 μg/mL) at 37 °C under aerobic conditions for 8 h, in the presence or absence of 10 mM H_3_PO_4_, 150 mM NaCl and/or 20 mM CaCl_2_. Next, the culture medium was carefully collected, and the adherent biofilms were gently washed twice with 200 μL of 100 mM sodium phosphate buffer (pH 7.4) to remove nonadherent planktonic cells for quantification. For nonadherent biofilm collection, the supernatants were centrifuged at 100×*g* at RT for 15 s. The collected nonadherent biofilms were gently washed twice with 200 μL of either 100 mM sodium phosphate buffer (pH 7.4) for biofilm quantification or BHI broth supplemented with 1 % sucrose for re-adhesion and reformation assays to remove planktonic cells. After each washing step, the samples were centrifuged at 100×*g* at RT for 15 s, and the nonadherent biofilm was retained. Vehicle-treated bacterial cultures served as the negative controls. Macroscopic images of the biofilms were digitally captured using a smartphone camera (iPhone 13 Pro) under standardized lighting conditions.

### 4.14 Immunofluorescence staining of S. mutans biofilms

*S. mutans* biofilms formed in a flat-glass-bottomed 16-well Lab-Tek chamber slide system were fixed by incubating with 4 % paraformaldehyde for 10 min. Each well was blocked with the Blocking One Histo blocking reagent for 10 min. For immunostaining, fB2Ms was visualized using a mouse anti-B2M AF polyclonal Ab as the primary Ab at RT for 60 min, followed by Alexa 488-conjugated anti-mouse IgG as the secondary Ab at RT for 30 min. Moreover, biofilms were visualized by staining with Alexa Fluor 594-conjugated ConA at RT for 60 min to label glycoconjugates on the biofilm surface and in regions of the biofilm matrix with lower density or reduced thickness. Subsequently, these biofilms were visualized by staining at RT for 10 min using Hoechst 33342 (Thermo Fisher Scientific, Cat# H3570), which is a cell-permeable, non-intercalating nuclear counterstain that emits blue fluorescence upon binding to AT-rich regions in the minor groove of genomic DNA in eukaryotic and prokaryotic cells. After each step, the slides were washed three times with PBS-T. All immunofluorescence assays were performed in the dark. Immunolabeled biofilms were mounted using the ProLong Glass Antifade Mountant. Fluorescence imaging was performed as described above using a Zeiss LSM 710 confocal laser scanning microscope equipped with a filter set for blue fluorescence (excitation, 405 nm; dichroic mirror, MBS 405; emission, 430 nm). For each sample, 10 fields of view were recorded in at least three independent experiments, and representative images were presented.

### 4.15 Assessment of bacterial growth by ATP production

The effect of fB2M on bacterial growth was evaluated by quantifying bacterial cell numbers based on ATP production. *S. mutans* was cultured to reach the logarithmic phase, and bacterial suspensions were prepared by diluting the cultures in fresh BHI broth to an OD_600_ of 0.025. Aliquots (120 μL) of these suspensions supplemented with 1 % (w/v) sucrose were prepared. Subsequently, 40 μL of a reaction mixture containing either vehicle (H_2_O), native B2M, or preformed fB2M (final protein concentration 100 μg/mL) was added to the bacterial suspensions. The cultures were incubated aerobically at 37 °C for 0, 2, 4, 6, or 8 h in 0.2-mL tubes (Watson Co., Cat# 137–211C), and bacterial growth profiles were determined using a BacTiter-Glo Microbial Cell Viability Assay Kit (Promega, Cat# G8230) according to the manufacturer's instructions. Bioluminescence was quantified using an Infinite M Plex multimode plate reader and expressed in terms of relative light units (RLU). Bacterial growth was calculated as follows: bacterial growth (%) = (Experimental RLU/Control RLU) × 100, where the control RLU values were obtained from vehicle-treated bacterial cultures at each time point. Bacterial growth was evaluated using at least three biological replicates.

### 4.16 Biofilm quantification

Biofilm bacteria were quantitatively analyzed using the crystal violet staining. Briefly, 100 μL of 0.1 % (w/v) crystal violet solution was added to each well to stain the adherent biomass and incubated for 30 min at RT. The excess crystal violet was aspirated, and the wells were rinsed twice with 200 μL of Milli-Q ultrapure water. Next, the plates were inverted and air-dried. Macroscopic images of the stained biofilms were digitally captured using a smartphone camera (iPhone 13 Pro) under standardized lighting conditions. To solubilize the bound crystal violet, 100 μL of 70 % (v/v) ethanol was added to each well at RT for 10 min, and 90 μL of the resulting solution was transferred to a 96-well white microtiter plate (Thermo Fisher Scientific, Cat# 236108). Subsequently, the absorbance was measured spectrophotometrically at 595 nm using an Infinite M Plex multimode plate reader. All growth conditions were evaluated using at least three biological replicates.

Furthermore, nonadherent biofilms were quantified. Briefly, the collected nonadherent biofilms were stained with 100 μL of a 0.1 % (w/v) crystal violet solution, followed by incubation for 30 min at RT and centrifugation at 100×*g* for 15 s. The excess crystal violet was carefully aspirated, and the biofilms were gently rinsed twice with 200 μL of Milli-Q ultrapure water. Following each washing step, the samples were centrifuged at 100×*g* at RT for 15 s, and the nonadherent biofilm mass was retained. The tubes were carefully inverted and air-dried. To solubilize the bound crystal violet, 100 μL of 70 % (v/v) ethanol was added to each well and incubated at RT for 10 min. Subsequently, 90 μL of the resulting solution was transferred to a 96-well white microtiter plate, and the absorbance was spectrophotometrically measured at 595 nm using an Infinite M Plex multimode plate reader. All growth conditions were evaluated using at least three biological replicates.

### 4.17 Re-adhesion and reformation assays of S. mutans biofilm

To assess the ability of nonadherent *S. mutans* biofilms to re-adhere, biofilms were initially formed in the presence of one of the following: 2.5 mM H_3_PO_4_ (vehicle control), B2M (100 μg/mL in 2.5 mM H_3_PO_4_), or preformed fB2M (100 μg/mL in 2.5 mM H_3_PO_4_). Nonadherent biofilm material in the supernatants (160 μL) were collected by centrifugation at 100×*g* and RT for 15 s. To remove planktonic cells, the resulting pellet was washed twice with 200 μL of BHI broth supplemented with 1 % sucrose.

The recovered nonadherent biofilms were resuspended in 160 μL of BHI broth supplemented with 1 % sucrose and transferred to individual wells of a new flat-glass-bottomed 16-well Lab-Tek Chamber Slide System. The samples were incubated at 37 °C under aerobic conditions for 8 h. Subsequently, re-adhered *S. mutans* biofilms were quantified as described in Section 4.16. All growth conditions were evaluated using at least three biological replicates.

To evaluate the capacity of nonadherent *S. mutans* biofilms to reform, the resuspended biofilm suspension was also diluted 10-fold with BHI broth supplemented with 1 % sucrose. From these dilutions, 160 μL aliquots were inoculated into individual wells of a new flat-glass-bottomed 16-well Lab-Tek Chamber Slide System. The wells were incubated at 37 °C under aerobic conditions for up to 8 h. Biofilm samples were collected at 0, 2, 4, 6, and 8 h to allow for a time-course analysis. The quantity of reformed, adherent *S. mutans* biofilms was measured as described in Section 4.16. All experimental conditions were evaluated using at least three biological replicates.

### 4.18 SEM

Biofilms of *S. mutans* formed after the incubation at 37 °C for 4 h were washed with 100 mM sodium phosphate buffer (pH 7.4), and fixed with 2.5 % glutaraldehyde (Nisshin EM, Cat# 3047) diluted by 100 mM sodium phosphate buffer (pH 7.4) at RT for 30 min, and washed twice with the same buffer. The samples were dehydrated using a graded ethanol series (50 %, 70 %, 80 %, 90 %, 95 %, and 99.5 % v/v), with each concentration applied twice sequentially, followed by a treatment with *tert*-butyl alcohol. The dehydrated specimens were freeze-dried, coated with osmium tetroxide via vapor deposition, and examined using a Hitachi S-4500 field-emission SEM instrument (Hitachi). Micrographs were acquired at magnifications of×5000 and × 30,000. For each sample, 10 fields of view were recorded in at least three independent experiments, and representative images were presented.

### 4.19 Statistical analysis

Data acquired from at least three separate experiments are expressed as mean ± standard deviation (SD). The significant difference between the two groups was analyzed using Welch's *t*-test. *P*-values of <0.001 or 0.01 were considered to indicate statistical significance. Not all statistically significant differences are presented in the graphs.

## CRediT authorship contribution statement

**Taiki Mori:** Writing – review & editing, Writing – original draft, Visualization, Validation, Methodology, Investigation, Funding acquisition, Data curation, Conceptualization. **Eisuke Domae:** Writing – review & editing, Validation, Methodology, Investigation, Data curation. **Mariko Hanaoka:** Validation, Methodology, Investigation. **Takeshi Into:** Writing – review & editing, Writing – original draft, Visualization, Supervision, Project administration, Investigation, Funding acquisition, Data curation, Conceptualization.

## Funding

This work was supported by Grants-in-Aid for Scientific Research (C: 25K13355 to T.M.; C: 25K13329 to E.D.; B: 25K02839 and C: 22K09937 to T.I.). Additional funding to T.M. was provided by a Miyata Grant for Scientific Research (A) from Asahi University, a grant from the 10.13039/100010742OGAWA Science and Technology Foundation, and a Koshiyama Research Grant. The funders had no role in the study design, data collection and analysis, decision to publish, or manuscript preparation.

## Declaration of competing interest

The authors declare that they have no known competing financial interests or personal relationships that could have appeared to influence the work reported in this paper.

## Data Availability

All relevant data are contained within the manuscript and supplementary data, additional data, if any, are available from the authors on appropriate request.

## Glossary

Ab: Antibody
AF: Amyloid fibril
B2M: β_2_-microglobulin
fB2M: Fibrillar β_2_-microglobulin
BHI: Brain heart infusion
ConA: Concanavalin A
RFU: Relative fluorescence unit
SCFA: Short chain fatty acid
SEM: Scanning electron microscopy
TEM: Transmission electron microscopy
ThT: Thioflavin T
