# Supplementary material for: Dental plaque biofilm transforms host-derived β2-microglobulin into polymorphic fibrils for integration into the biofilm matrix
Source: Biofilm. 2025 Nov 16;10:100331. doi: 10.1016/j.bioflm.2025.100331 (PMC12666850; doi:10.1016/j.bioflm.2025.100331)
Supplement: Multimedia component 1 [file mmc1.pdf]

Supplementary Materials

Dental plaque biofilm transforms host-derived β_2-microglobulin into polymorphic fibrils for integration into the biofilm matrix

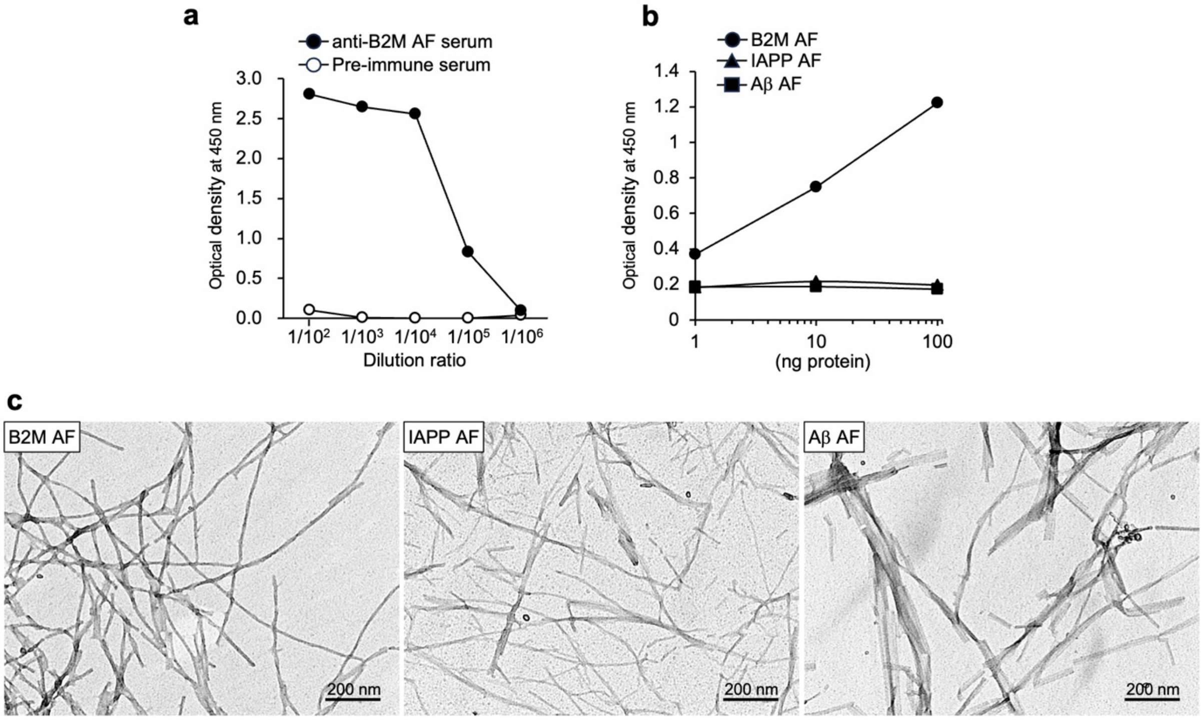

Fig. S1. Determination of the titer and specificity of the anti-β_2-microglobulin amyloid fibril (B2M AF) antibody (Ab) using enzyme-linked immunosorbent assay (ELISA). (a) Comparative immunoreactivity in the antiserum against B2M AF collected 70 days post-immunization and pre-immune serum. B2M AF (0.1 μg) coated on ELISA plates was reacted with 10-fold serially diluted anti-B2M AF serum or pre-immune serum. **(b)** Cross-reactivity of the anti-B2M AF polyclonal Ab with well-characterized AFs derived from heterologous peptides. Increasing doses of B2M AF, human islet amyloid polypeptide (IAPP) AF, and human amyloid β (1-42) peptide (Aβ) AF were tested. Ten-fold serial dilutions of B2M AF, IAPP AF, or Aβ AF coated on ELISA plates were treated with diluted anti-B2M AF Ab (1:10,000). **(c)** Transmission electron microscopy (TEM) images of B2M AF formed in the presence of 10 mM HCl with 150 mM NaCl (left), IAPP AF formed in phosphate buffered saline (PBS) (middle), and Aβ AF formed in PBS (right). Each TEM image was captured at a magnification of ×25,000. Representative images acquired from at least three independent experiments are shown. Scale bars: 200 nm.

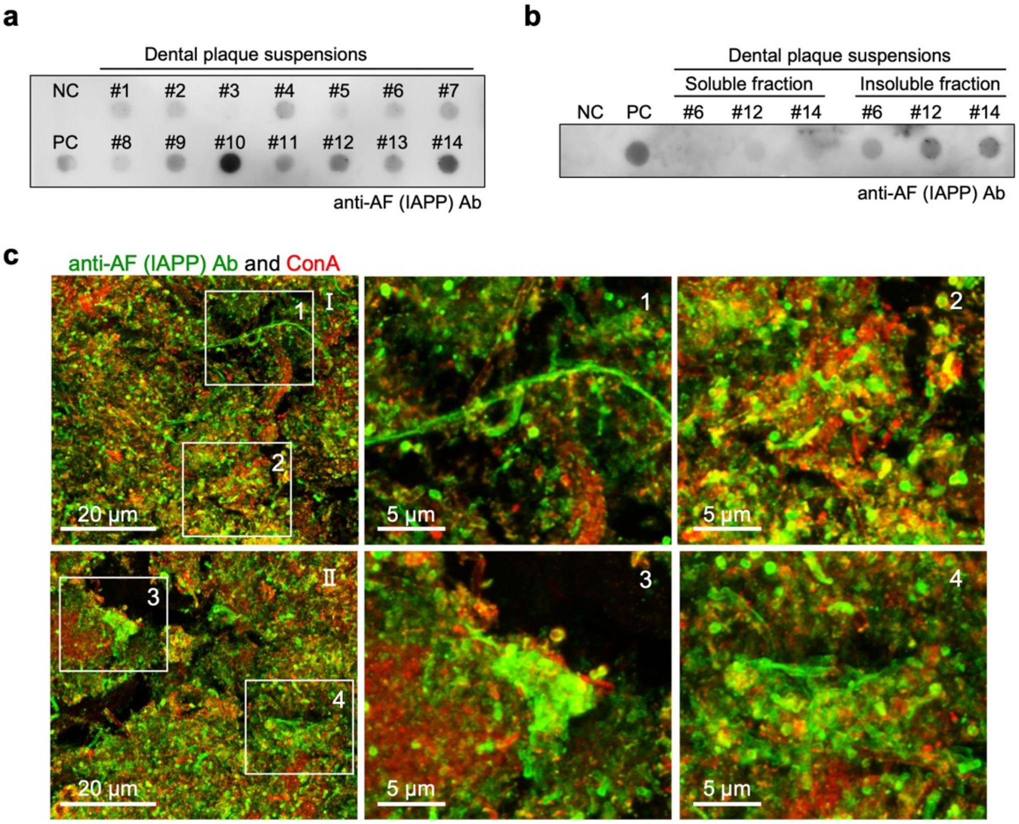

Fig. S2. Immunological assay of dental plaque-associated fibrils using an anti-amyloid specific antibody (Ab). (a) Dot blot analysis of fibrils in dental plaque suspensions acquired from 14 participants (Table S3) using an anti-amyloid fibril (AF) Ab raised against AFs derived from human islet amyloid polypeptide (IAPP). NC, negative control (phosphate buffered saline, PBS); PC, positive control (1 μg fibrillized IAPP). **(b)** Dot blot analysis of fibrils in the soluble and insoluble fractions of three dental plaque suspensions (#6, #12, and #14) using the anti-AF (IAPP) Ab. NC, negative control (PBS); PC, positive control (0.2 μg fibrillized IAPP). **(c)** Confocal microscopy images of dental plaque specimens immunostained with an anti-AF (IAPP) Ab followed by Alexa Fluor 488-conjugated anti-rabbit IgG (green) and counterstained with Alexa Fluor 594-conjugated concanavalin A (ConA; red) for extracellular glycoconjugates. Scale bars: 20 μm. Images I and II (upper and lower left) depict different views of dental plaque specimens. Image 1 shows a magnified view of inset 1 in view I (white box, upper left image), which exhibits an abundance of fibrils with varying fibrillar morphologies. Image 2 shows a magnified view of inset 2 in view I (white box, upper left image), exhibiting an abundance of fibrils with punctate structures of various sizes. Image 3 shows a magnified view of inset 3 in view II (white box, lower left image) exhibiting amorphous aggregates of fibrils. Image 4 shows a magnified view of inset 4 in view II (white box, lower left image) exhibiting fibrils with short fibrillar morphologies. Scale bars: 5 μm.

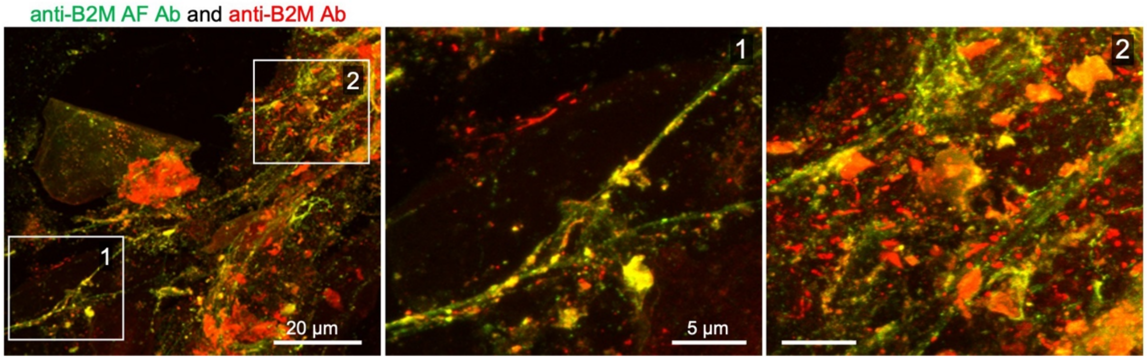

Fig. S3. Co-immunological detection of fibrillar β_2-microglobulin (B2M) and B2M in dental plaque. Immunofluorescence staining with an anti-B2M amyloid fibril (B2M AF) antibody (Ab) (green) and an anti-B2M Ab (red) followed by confocal microscopy analysis of dental plaque specimens. Scale bar: 20 µm. The areas outlined in white (1 and 2) in the left image are magnified and displayed in (1) (middle) and (2) (right). Scale bars: 5 µm. Representative images from at least three independent experiments are presented.

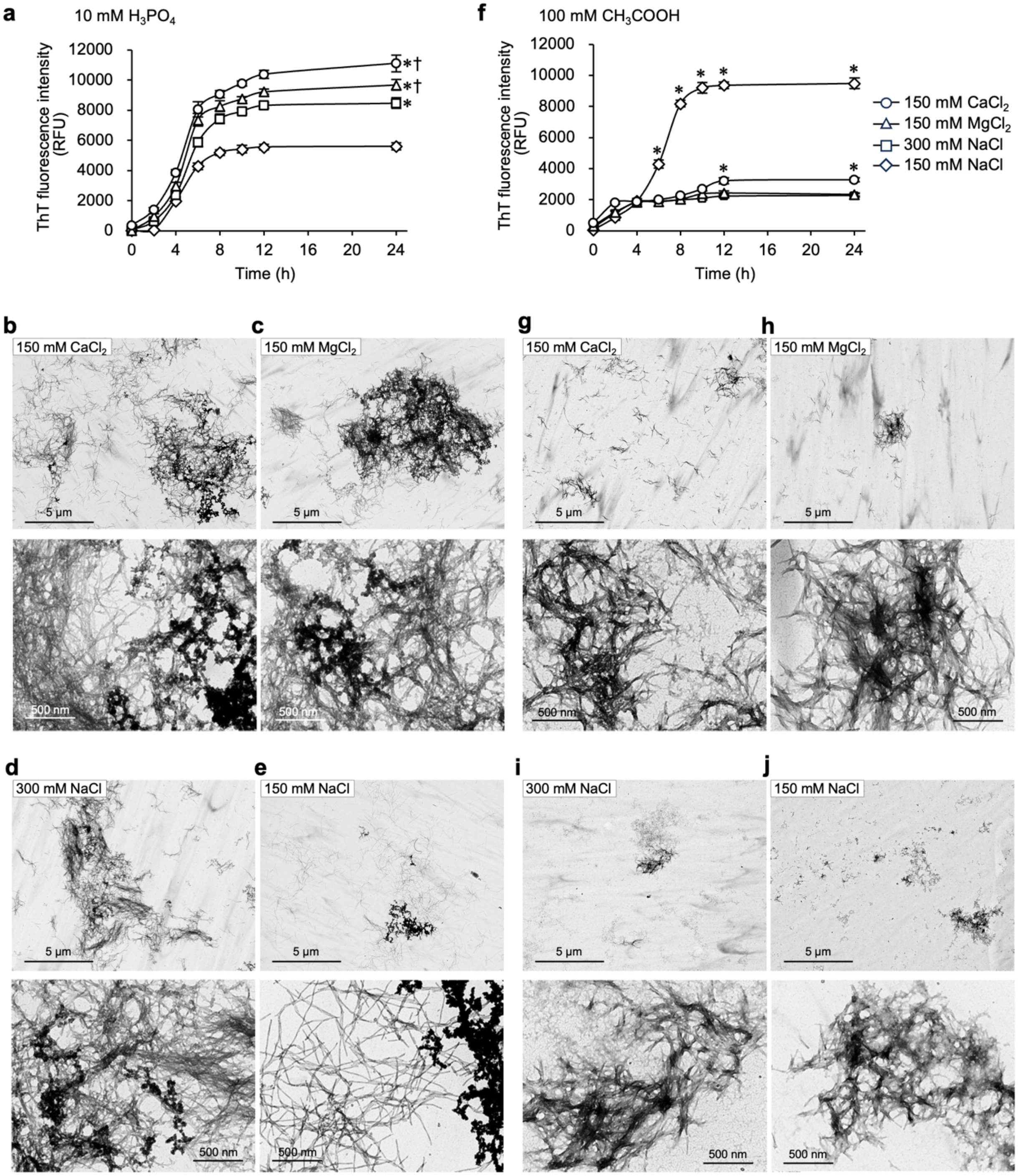

a
10 mM H3PO4
ThT fluorescence intensity (RFU)
Time (h)
f
100 mM CH3COOH
150 mM CaCl2
150 mM MgCl2
300 mM NaCl
150 mM NaCl
b
150 mM CaCl2
c
150 mM MgCl2
g
150 mM CaCl2
h
150 mM MgCl2
d
300 mM NaCl
e
150 mM NaCl
i
300 mM NaCl
j
150 mM NaCl
5 μm
500 nm

Fig. S4. Fibrillar β_2-microglobulin (fB2M) formation is significantly influenced by divalent cations. (a) Effect of divalent cations (Ca^{2+} and Mg^{2+}) on fB2M formation under the acidic conditions induced by H_3PO_4. B2M (500 μg/mL) was incubated at 37 °C for 0–24 h with 150 mM $CaCl_2$ (open circle), 150 mM $MgCl_2$ (open triangle), 300 mM NaCl (open square), or 150 mM NaCl (open diamond) in the presence of 10 mM H_3PO_4. Thioflavin T (ThT) fluorescence intensity (relative fluorescence units, RFU) reflecting fB2M formation is presented as the mean ± SD (n ≥ 3). $^{*}P < 0.01$ compared to 150 mM NaCl at 24 h. $^{\dagger}P < 0.01$ compared to 300 mM NaCl at 24 h. **(b–e)** Transmission electron microscopy (TEM) images of B2M treated with 150 mM $CaCl_2$ (b), 150 mM $MgCl_2$ (c), 300 mM NaCl (d), or 150 mM NaCl (e) in the presence of 10 mM H_3PO_4. Images were acquired at magnifications of ×2,000 (upper) and ×15,000 (lower). Scale bars: 5 μm and 500 nm, respectively. **(f)** Effect of divalent cations (Ca^{2+} and Mg^{2+}) on fB2M formation under the acidic conditions induced by CH_3COOH. B2M (500 μg/mL) was incubated at 37 °C for 0–24 h with 150 mM $CaCl_2$ (open circle), 150 mM $MgCl_2$ (open triangle), 300 mM NaCl (open square), or 150 mM NaCl (open diamond) in the presence of 100 mM CH_3COOH. ThT fluorescence intensity (RFU) reflecting fB2M formation is presented as the mean ± SD (n ≥ 3). $^{*}P < 0.01$ compared to 300 mM NaCl at each time. **(g–j)** TEM images of B2M treated with 150 mM $CaCl_2$ (g), 150 mM $MgCl_2$ (h), 300 mM NaCl (i), or 150 mM NaCl (j) in the presence of 100 mM CH_3COOH. Images were acquired at magnifications of ×2,000 (upper) and ×15,000 (lower). Scale bars: 5 μm and 500 nm, respectively.

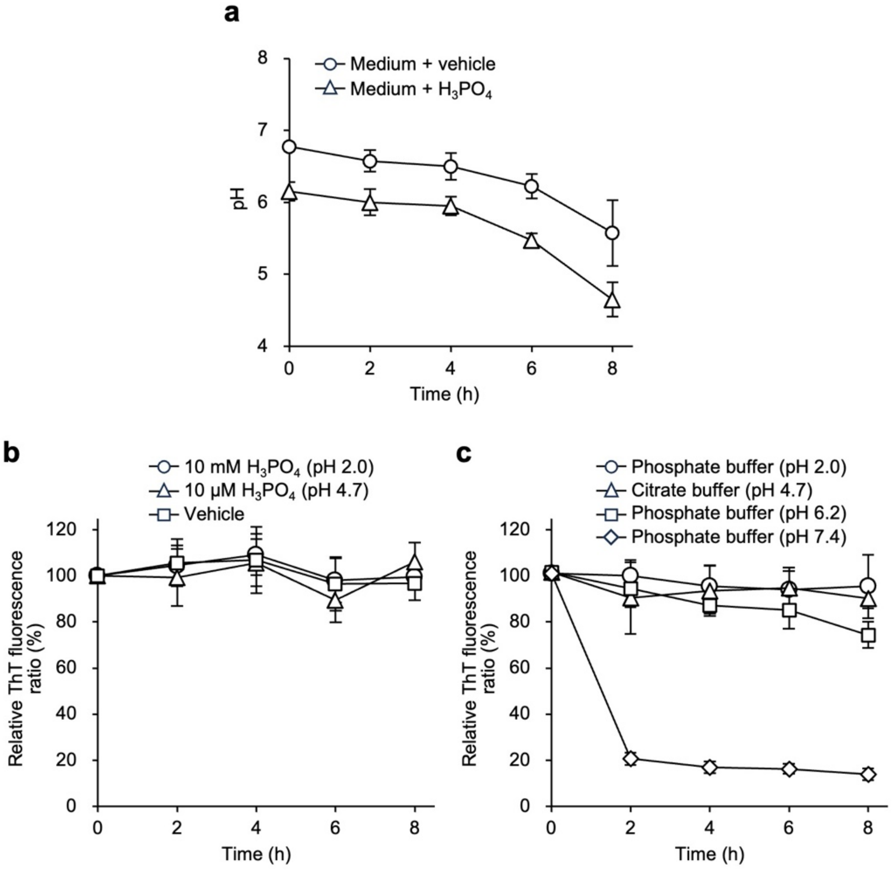

Fig. S5. Relationship between pH changes in *Streptococcus mutans* biofilm formation medium and fibrillar β$_2$-microglobulin (fB2M) stability. (a) Time course of pH changes in *S. mutans* biofilm formation medium. *S. mutans* in brain heart infusion (BHI) broth containing 1% sucrose was incubated at 37 °C for 0–8 h with vehicle (H_2O) or 10 mM H_3PO_4; pH values are presented as the mean ± SD ($n \geq 3$). **(b)** fB2M stability in phosphoric acid solutions at different pH values. fB2Ms (500 µg/mL) were incubated at 37 °C for 0–8 h in 10 mM phosphoric acid (pH 2.0), 10 µM phosphoric acid (pH 4.7), or vehicle (H_2O) with 150 mM NaCl. The relative Thioflavin T (ThT) fluorescence ratio (% vs. ThT fluorescence intensity at 0 h) is presented as the mean ± SD ($n \geq 3$). **(c)** fB2M stability in buffers of different pH values. fB2Ms (500 µg/mL) were incubated at 37 °C for 0–8 h in 20 mM sodium phosphate buffer (pH 2.0), 20 mM sodium citrate buffer (pH 4.7), 20 mM sodium phosphate buffer (pH 6.2), or 20 mM sodium phosphate buffer (pH 7.4) with 150 mM NaCl. The relative ThT fluorescence ratio (% vs. ThT fluorescence intensity at 0 h) is presented as the mean ± SD ($n \geq 3$).

Table S1. Major salivary proteins (adapted from Amado *et al.*[*])

No.	Protein name	No.	Protein name
1	Albumin	26	Lysozyme
2	Amylase	27	Matrix Metallopeptidase-1
3	C-reactive protein	28	Matrix Metallopeptidase-2
4	C3 Complement	29	Matrix Metallopeptidase-3
5	C4 Complement	30	Matrix Metallopeptidase-9
6	CA15-3	31	Mucin 5
7	Carbonic anhydrase VI	32	Mucin 7
8	Cathepsin G	33	Myoglobin
9	Cathepsin L	34	Neutrophil elastase
10	Complement Factor B	35	Osteopontin
11	Cystatins (SN, SA, S, C, D)	36	Plasminogen
12	Fibronectin	37	Proline-rich proteins
13	GP340	38	S100 proteins (A7, A8, A9, B)
14	Haptoglobin	39	SIgA
15	Histatins (1, 3, 5)	40	Statherin
16	HNP1-3 (α-Defensins)	41	Thymosin
17	IgE	42	TIMP Metallopeptidase Inhibitor 1
18	IgG	43	TIMP Metallopeptidase Inhibitor 2
19	Interleukin-1α	44	Transferrin
20	Interleukin-1β	45	Troponin 1
21	Interleukin-2	46	Tumor necrosis factor α
22	Interleukin-6	47	α_1-Antitrypsin
23	Interleukin-8	48	α_2-Macroglobulin
24	Lactoferrin	49	β-Defensins (hBD-2, -3)
25	LL-37	50	β_2-Microglobulin

[*]Reference

Amado FM, Ferreira RP, Vitorino R. One decade of salivary proteomics: current approaches and outstanding challenges. Clin. Biochem. 2013;46:506–517. https://doi.org/10.1016/j.clinbiochem.2012.10.024.

Table S2. Amyloidogenic proteins (adapted from Chiti *et al.*[*])

No.	Protein name	Associated disease
1	ABri peptide	Familial British dementia
2	ADan peptide	Familial Danish dementia
3	Apolipoprotein C-II	ApoCII amyloidosis
4	Apolipoprotein C-III	ApoCIII amyloidosis
5	Atrial natriuretic factor	Atrial amyloidosis
6	C-term fragments of kerato-epithelin	Lattice corneal dystrophy type 1,<br>Lattice corneal dystrophy type 3A,<br>Lattice corneal dystrophy Avellino type
7	C-terminally extended Apolipoprotein AII	ApoAII amyloidosis
8	Calcitonin	Medullary carcinoma of the thyroid
9	Corneodesmosin	Hypotrichosis simplex of the scalp
10	Enfuvirtide (compound)	Injection-localized amyloidosis
11	Fragments of fibrinogen α-chain	Fibrinogen amyloidosis
12	Fragments of gelsolin	Familial amyloidosis, Finnish type
13	Fragments of immunoglobulin heavy chains	Heavy chain amyloidosis
14	Fragments of immunoglobulin light chains	Light chain amyloidosis
15	Full length of N-terminal fragments of serum amyloid A protein	AA amyloidosis
16	Galectin-7	Lichen amyloidosis, Macular amyloidosis
17	Huntingtin exon 1	Huntington's disease
18	Insulin	Injection-localized amyloidosis
19	Islet amyloid polypeptide (IAPP)	Diabetes mellitus type 2, Insulinoma
20	Lactadherin / Medin (Milk fat globule EGF and factor V/VIII domain containing)	Aortic medial amyloidosis
21	Lactotransferrin / Lactoferrin	Gelatinous drop-like corneal dystrophy
22	Leukocyte cell-derived chemotaxin-2	Renal amyloidosis
23	Lysozyme	Lysozyme amyloidosis
24	Microtubule-associated protein tau	Various forms of tauopathies<br>(e.g., Pick's disease, Progressive supranuclear palsy, Corticobasal degeneration, Frontotemporal dementia with parkinsonism linked to chromosome 17, Argyrophilic grain disease)
25	N-term fragments of prolactin	Pituitary prolactinoma
26	N-terminal fragments of apolipoprotein AI	ApoAI amyloidosis
27	N-terminal fragments of apolipoprotein AIV	ApoAIV amyloidosis
28	N-terminally truncated cystatin C	Hereditary cerebral hemorrhage with amyloidosis, Icelandic type
29	Odontogenic ameloblast-associated protein	Calcifying epithelial odontogenic tumors
30	Prion protein	Transmissible spongiform encephalopathy (e.g., Fatal familial insomnia, Gerstmann-Sträussler-

		Scheinker disease, Creutzfeldt-Jacob disease, New variant Creutzfeldt-Jacob disease)
31	Pulmonary surfactant-associated protein C	Pulmonary alveolar proteinosis
32	S100A8/A9 proteins	Prostate cancer
33	Semenogelin-1	Seminal vesicle amyloidosis
34	Transthyretin	Senile systemic amyloidosis, Familial amyloid polyneuropathy, Familial amyloid cardiomyopathy, Leptomeningeal amyloidosis
35	α-Synuclein	Parkinson's disease, Parkinson's disease dementia, Dementia with Lewy bodies, Multiple system atrophy
36	B Amyloid peptide (Aβ) from amyloid precursor protein	Alzheimer's disease, Hereditary cerebral hemorrhage with amyloidosis
37	β_2-Microglobulin	Dialysis-related amyloidosis, Hereditary visceral amyloidosis

*Reference

Chiti F, Dobson CM. Protein misfolding, amyloid formation, and human disease: A summary of progress over the last decade. Annu. Rev. Biochem. 2017;86:27–68. https://doi.org/10.1146/annurev-biochem-061516-045115.

Table S3. Participants' information

#	Age	Sex
1	54	F
2	56	F
3	36	M
4	35	F
5	42	F
6	48	F
7	47	F
8	16	M
9	53	M
10	44	F
11	49	M
12	42	F
13	55	M
14	42	F

M, male; F, female
